# Effects of acceptance and commitment therapy on psychological and health-related outcomes in stroke patients: a systematic review and meta-analysis of randomized controlled trials

**DOI:** 10.3389/fneur.2026.1953111

**Published:** 2026-10-28

**Authors:** Xiaohuan Li, Weijin Huang, Ying Gao, Yu'e Wu, Ping Wang

**Affiliations:** 1 School of Nursing, Guangdong Medical University, Dongguan, Guangdong, China; 2 Department of Gastrointestinal Infection, Houjie Clinical College of Guangdong Medical University, Houjie Hospital, Dongguan, Guangdong, China; 3 Department of Neurology, Houjie Clinical College of Guangdong Medical University, Houjie Hospital, Dongguan, Guangdong, China

**Keywords:** acceptance and commitment therapy, anxiety, depression, meta-analysis, psychological flexibility, stroke rehabilitation

## Abstract

**Background:**

Stroke is a leading cause of disability worldwide and is frequently accompanied by psychological and functional impairments. Acceptance and Commitment Therapy (ACT), a third-wave behavioral intervention focusing on psychological flexibility, has been increasingly applied in stroke rehabilitation. Although previous reviews have suggested beneficial effects of ACT in stroke patients, whether these benefits persist in the short term after the intervention ends, and the certainty of the evidence, remain unclear.

**Methods:**

A systematic review and meta-analysis was conducted according to PRISMA guidelines and registered in PROSPERO (CRD420261325108). Randomized controlled trials evaluating ACT in stroke patients were included. Pooled effects were calculated using random-effects models, with separate analyses for post-intervention and follow-up outcomes. Sources of heterogeneity were explored using subgroup analyses and meta-regression. Risk of bias was assessed using the Cochrane Risk of Bias 2 tool, and certainty of evidence was evaluated using the GRADE framework.

**Results:**

Twenty-two RCTs involving 1,975 participants were included. ACT may be associated with reductions in depressive symptoms (post-intervention: SMD = −1.32, 95% CI: −1.80 to −0.84; follow-up: SMD = −0.76, 95% CI: −1.07 to −0.45) and anxiety symptoms (post-intervention: SMD = −1.36, 95% CI: −1.90 to −0.82; follow-up: SMD = −0.51, 95% CI: −1.42 to 0.40, not significant); depressive symptoms remained statistically significant at follow-up. Heterogeneity was considerable for both outcomes at post-intervention (*I^2^* = 93.68% for depression and 93.60% for anxiety), so the pooled estimates represent average effects across studies. ACT may also be associated with improvements in psychological flexibility, quality of life, and sleep quality; however, these outcomes were based on a limited number of studies and showed considerable heterogeneity (*I^2^* = 74.68 to 92.26%), so they should be interpreted cautiously. However, no statistically significant effects were observed for activities of daily living, neurological function, or hope. ACT did not appear to increase dropout rates in the available studies (*K* = 16; RR = 0.99, 95% CI: 0.58–1.69). Meta-regression analyses indicated that intervention duration may not be a significant moderator of treatment effects. The certainty of evidence ranged from very low to moderate, mainly due to risk of bias and inconsistency.

**Conclusion:**

Acceptance and Commitment Therapy may be a useful adjunctive intervention for improving psychological outcomes in people with stroke. However, evidence for functional recovery remains insufficient. Future high-quality RCTs with standardized protocols, larger samples, and longer follow-up are needed to confirm the long-term effectiveness of ACT.

**Systematic review registration:**

https://www.crd.york.ac.uk/PROSPERO/, identifer CRD420261325108.

## 1 Background

Stroke is defined as a sudden onset of focal neurological deficit lasting ≥24 h or associated with acute brain imaging evidence of infarction or hemorrhage (1). Stroke is a leading cause of death and disability worldwide (1). Many survivors are left with persistent disability: in a large cohort of patients with ischemic stroke, approximately 40% had moderate-to-severe or persistent severe disability during the first year (2). Post-stroke depression affects roughly 10–35% of patients during the first 3 months (3), and anxiety affects about one in four patients when assessed with rating scales (4); anxiety after stroke has been described as the “other” poststroke mood disorder (5). Depression is associated with poorer functional outcomes over the first 5 ears after stroke (3), and at 6 months, with cognitive impairment (6). These symptoms are often not transient: long-term cohort data show that a considerable proportion of survivors still report elevated depressive and anxiety symptoms several years after stroke, and that these rates are relatively stable over time (7).

Current management of post-stroke psychological distress relies mainly on pharmacological treatment and general psychosocial or rehabilitation support. Antidepressants are widely prescribed, but a Cochrane review found that the evidence for pharmacological and psychological interventions for depression after stroke is of very low certainty, and that antidepressants were associated with adverse events involving the central nervous system and the gastrointestinal tract (8). At present, traditional intervention methods such as cognitive behavioral therapy (CBT) have been shown to reduce depressive and anxiety symptoms after stroke, with large effects at the end of treatment and moderate maintenance at 3 months, although the evidence comes from a small number of generally small studies (9). Psychological treatment is recommended in stroke care (10).

Acceptance and Commitment Therapy (ACT) is the third wave of behavioral intervention, and it has gained increasing attention due to its focus on psychological flexibility (11). ACT emphasizes psychological flexibility, helping individuals accept distressing experiences and engage in value-based behaviors despite ongoing health challenges. This approach may be particularly relevant for stroke survivors who face persistent functional limitations and emotional distress during recovery (11). Rather than attempting to reduce or control distressing thoughts and feelings, ACT seeks to increase psychological flexibility — the capacity to remain in contact with the present moment and to persist in, or adjust, behavior in the service of personally meaningful values — through acceptance, cognitive defusion, and committed action (11). This process-oriented approach may be particularly relevant after stroke, where disability and uncertainty about recovery are often not modifiable: cognitive fusion has been found to predict depressive symptoms and to mediate the association between psychological flexibility and depression, suggesting that cognitive defusion may be a relevant treatment target (12). Previous evidence has primarily focused on immediate post-intervention outcomes, while the durability of ACT effects after intervention remains unclear (13). A recent systematic review and meta-analysis reported that ACT improved depression, anxiety, and quality of life in stroke patients (13). Evidence from other populations is mixed: a randomized trial in people with acquired brain injury found no superiority of ACT over an active control on the primary outcomes, although more participants showed long-term, individual-level improvement in depressive and anxiety symptoms (14); benefits have been reported in older adults with chronic low back pain (15) and in people with type 2 diabetes (16), and internet-delivered ACT has been associated with reduced anxiety (17). Whether the benefits of ACT are maintained after the intervention ends, and which outcomes and patient groups respond most consistently, therefore remain unclear.

To address these uncertainties, this systematic review and meta-analysis aimed to provide an updated and comprehensive evaluation of ACT in stroke patients. Specifically, we evaluated the effects of ACT on psychological outcomes (depression, anxiety, and ACT-related psychological processes, including psychological flexibility and cognitive fusion), clinical outcomes (quality of life, sleep quality, activities of daily living, and neurological function), and all-cause attrition (used as an indirect indicator of participant retention rather than as a direct measure of acceptability). We also examined whether the effects of ACT were maintained at follow-up, explored potential sources of heterogeneity through subgroup analyses and meta-regression, and assessed the certainty of evidence using the GRADE framework. By integrating updated evidence from randomized controlled trials, this review aims to clarify the role of ACT in stroke rehabilitation and to inform future clinical practice and research.

## 2 Methods

This meta-analysis followed the Preferred Reporting Items for Systematic Reviews and Meta-Analyses (PRISMA) guidelines. The study protocol was prospectively registered in PROSPERO (CRD420261325108) on 24 February 2026, and the PRISMA checklist is provided in Appendix 1. No amendments were made to the eligibility criteria or primary outcomes; the final search was updated to 6 March 2026 to ensure coverage of the most recent literature.

### 2.1 Search strategy

We conducted a comprehensive literature search across eight English-language databases (PubMed, MEDLINE [via Ovid], Embase [via Ovid], Cochrane Library, CINAHL, Web of Science, Scopus, and PsycINFO) and four Chinese databases (CNKI, Wanfang, SinoMed, VIP) from their inception to March 6, 2026. Reference lists of included studies and relevant reviews were manually examined, and the Journal of Contextual Behavioral Science was screened for additional eligible RCTs. The search combined Medical Subject Headings (MeSH) with free-text terms, including “Acceptance and Commitment Therapy,” “stroke,” “Apoplexy,” and “Cerebrovascular Accident*.” Detailed search strategies for each database are included in Appendix 2.

### 2.2 Eligibility criteria

Selection of studies was based on the PICOS framework. Participants (P): Adults diagnosed with stroke (ischemic or hemorrhagic). Interventions (I): ACT-based interventions delivered in any format (face-to-face, group, tele-rehabilitation, or online). An intervention was classified as ACT-based if the authors explicitly described it as ACT and it was delivered as a structured program following the six core ACT processes by a named facilitator; single-technique or generic third wave/mindfulness interventions without an explicit ACT rationale were excluded. Comparisons (C): Conventional care without ACT, including medical treatment, routine nursing, traditional psychotherapy, rehabilitation training, health education, or other standard care. Outcomes (O): Primary: Depression and anxiety. Secondary: Psychological processes (psychological flexibility, cognitive fusion), clinical outcomes (quality of life, sleep quality, activities of daily living, neurological function), and positive psychological resources (self-efficacy, hope, and resilience). Attrition: dropout rates. Only outcomes measured with validated instruments (e.g., PHQ-9, HADS, AAQ-II, SS-QOL, PSQI, and BI) were considered. Study Design (S): Randomized controlled trials (RCTs).

For studies with multiple reports, the publication with the most complete dataset or largest sample size was selected. Outcomes were classified as post-intervention or follow-up, with separate meta-analyses performed for each time point to evaluate the durability of ACT effects.

### 2.3 Study selection and data extraction

Records were imported into NoteExpress 2018, and duplicates removed (Y’E). Two independent reviewers (XH and WJ) screened titles and abstracts, followed by full-text assessment of potentially relevant articles. Disagreements were resolved through discussion or consultation with a third reviewer (GY). Data extraction employed a structured form capturing authorship, publication year, sample size, stroke subtype, intervention and control details, and outcome measures. Inter-rater agreement was quantified using Cohen’s kappa coefficient. Medians and interquartile ranges were transformed into means and standard deviations using published methods (18). For studies with missing or unclear information, attempts were made to contact the original authors. All outcome measures were harmonized prior to analysis to ensure consistency across studies.

### 2.4 Risk of bias assessment

The Cochrane Risk of Bias tool version 2 (RoB 2) was used to evaluate methodological quality. Two reviewers (XH and WJ) independently assessed five domains: (1) randomization process, (2) deviations from intended interventions, (3) missing outcome data, (4) outcome measurement, and (5) selection of reported results. Each study was rated as low risk, some concerns, or high risk of bias. Discrepancies were resolved via discussion or consultation with a third reviewer (GY). Inter-rater reliability was determined using Cohen’s kappa coefficient. For Domain 5, as no trial had a publicly available protocol, reported outcomes were compared with each paper’s own Methods; absence of registration alone was not sufficient for high risk. Overall risk of bias was rated high only when at least one domain was rated high risk.

### 2.5 Data synthesis and statistical analysis

#### 2.5.1 Effect size and statistical model

Meta-analyses were conducted using Stata 18.0. Continuous outcomes were analyzed using mean differences (MDs) or standardized mean differences (SMDs) with 95% confidence intervals (CIs), depending on measurement consistency. Dichotomous outcomes, including dropout, were analyzed using relative risks (RRs) with 95% CIs. Effect sizes were interpreted according to Cohen’s criteria (0.2 = small, 0.5 = medium, 0.8 = large). A random-effects model with the DerSimonian-Lairdτ^2^ estimator was applied for pooled estimates; meta-regression and trim-and-fill used restricted maximum likelihood (REML). Statistical significance was set at *p* < 0.05 (two-sided). In this review, short-term follow-up was defined as outcome assessment performed within 3 months after the end of the intervention. Post-intervention and follow-up outcomes were analyzed separately. Meta-regression was restricted to outcomes with at least 10 studies per covariate; depression (*K* = 13) and anxiety (*K* = 10) met this criterion, while the AAQ-II model (*K* = 7) was exploratory only.

#### 2.5.2 Heterogeneity, subgroup, and sensitivity analyses

Heterogeneity was evaluated using Cochran’s Q test and the I^2^ statistic, interpreted as low (25%), moderate (50%), or high (75%). Q test significance was set at *p* < 0.10. Subgroup analyses and meta-regression analyses were conducted, when sufficient data were available, to explore potential sources of heterogeneity. Subgroup analyses were performed for predefined variables, with between-subgroup differences tested by Cochran’s Q; strata containing a single study were reported descriptively only. Intervention duration was entered as a raw continuous covariate (in weeks), without centering or standardization. Sensitivity analyses employed a leave-one-out approach to assess the robustness of the pooled estimates.

#### 2.5.3 Publication bias and certainty of evidence

Publication bias was assessed using funnel plots and Egger’s regression test for outcomes with ≥10 studies. A two-sided *p* < 0.1 indicated potential bias. When publication bias was suggested, the trim-and-fill method was additionally applied to examine the potential impact of missing studies on pooled estimates. The certainty of evidence for key outcomes was evaluated using the GRADE framework and summarized in a Summary of Findings table.

## 3 Results

### 3.1 Study selection

A total of 449 records were retrieved from the database, and 20 records were retrieved from the *Journal of Contextual Behavioral Science*. A total of 158 duplicate records were excluded. After screening titles and abstracts, an additional 276 studies were excluded. Upon reviewing the full texts of the remaining 35 studies, 13 were excluded because they were not randomized controlled trials (RCTs) (*n* = 2), the study participants did not meet the eligibility criteria (*n* = 3), they were duplicate reports of the same study (*n* = 5), not adults (participants aged <18 years) (*n* = 1), or the publication type was ineligible (*n* = 2). Ultimately, 22 studies were included. The PRISMA flowchart is shown in Figure 1.

**Figure 1:**
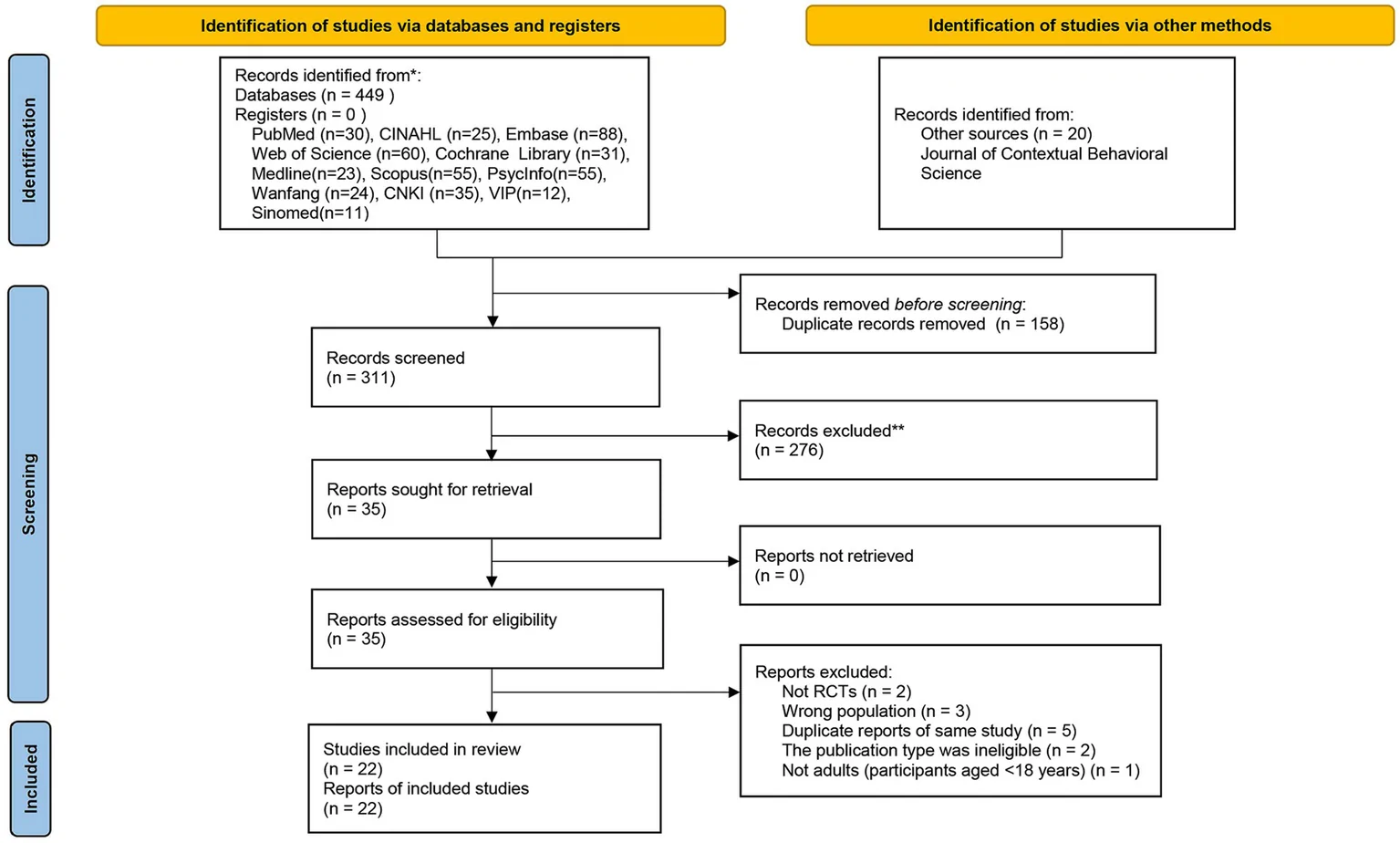
PRISMA flowchart.

### 3.2 Study characteristics

The 22 included RCTs enrolled 1,975 participants, predominantly from China, with sample sizes ranging from 53 to 140. ACT was delivered either alone or alongside usual care or routine rehabilitation. Intervention duration ranged from 1 to 12 weeks, primarily via face-to-face sessions, conducted individually or in groups. ACT was most commonly delivered by nurses (13 of the 22 trials), followed by psychological therapists. Control conditions typically comprised usual care and standard rehabilitation. Most studies assessed outcomes at baseline and immediately post-intervention, with fewer reporting follow-up data. Frequently measured outcomes included depression, anxiety, psychological flexibility, cognitive fusion, quality of life, sleep quality, and activities of daily living. Reporting of stroke subtype was inconsistent. In this review, short-term follow-up refers to outcome assessment performed within 3 months after the end of the intervention. The main characteristics of the included studies are shown in Table 1.

**Table 1:** Characteristics of the included studies.

Author (year)	Sample size (I/C)	Stroke subtype	Age (years) (I/C)	Interventions		Total duration/session/session duration	ACT provider	Format/medium	Assessment points	Outcome (measures)
				Experimental	Control					
Liu (2023) (35)	70/70	NR	62.21 ± 9.937/61.61 ± 10.843	ACT	Usual care	4w/7session/45-60 min	Nurses	Group/Face-to-face	Baseline, post-intervention, 3 m follow-up	HAMD-24, HRQoL-12, PSQI, AAQ-II, CFQ-9, CaSM-27
Wang (2025) (36)	60/60	NR	68.57 ± 5.36/68.07 ± 4.23	ACT	Usual care	12w/12session/60 min	ACT therapists	NR/Face-to-face	Baseline, post-intervention	AAQ-II, CFQ, HADS-A, HADS-D, ESDQ, Serum biomarkers
Niu (2022) (31)	52/52	Ischemic	61.5 ± 11.5/64.8 ± 12.1	ACT	Usual care	2w/5session/45-55 min	Psychological therapist and Nurses	Group/Face-to-face and telephone	Baseline, post-intervention, 1 m follow-up, 3 m follow-up	HAMD-24, NIHSS, BI
Majumdar (2019) (22)	26/27	NR	65.3 ± 11.9/60.0 ± 15.6	ACT	Usual care	4w/4session/120 min	Multi-disciplinary team^1^	Group/Face-to-face	Baseline, post-intervention, 2 m follow-up	PHQ-9, GAD-7, EQ-5D-5L Index Value, EQ VAS, AHS, WEMWBS
Wang (2022) (37)	50/50	Ischemic	62.04/60.86(SD NR)	ACT	Usual care	4w/7session/NR	Nurses	NR/Face-to-face	Baseline, post-intervention	AAQ-II, CFQ, SAS, SDS, PSQI, AIS
Zhang (2026) (38)	41/41	NR	NR	ACT	Usual care	12w/NR/NR	Medical staff	NR/NR	Baseline, post-intervention	PSQI, NFI-Stroke
Lai (2025) (39)	59/59	Mixed	55.49 ± 4.05/55.32 ± 5.11	ACT	Usual care	4w/8-12session/30-40 min	Psychological therapist and Nurses	Individual/Face-to-face	Baseline, post-intervention	FoP-Q-SF, Coping Style Questionnaire (62 items), HHI, Nursing satisfaction*
Sun (2024) (40)	30/30	Mixed	56.56 ± 1.82/56.58 ± 1.32	ACT	Usual care	NR/NR/60 min	Nurses	NR/Face-to-face	Baseline, post-intervention	SAS, SDS, CD-RISC, SS-QOL
Jia (2023) (41)	30/30	Ischemic	59.70 ± 11.38/62.07 ± 10.43	ACT	Usual care	2w/5session/30-40 min	Nurses	Individual/Face-to-face	Baseline, post-intervention	PSQI, HADS (A/D), AAQ-II, SS-QOL
Fu (2025) (19)	38/38	Ischemic	NR/ NR	ACT	Usual care	12w/NR/45-60 min	Nurses	Group/Face-to-face and online	Baseline, post-intervention, 1 m follow-up, 3 m follow-up	SSCI, CD-RISC-10, AAQ-II, CFQ-F
Wang (2024) (42)	46/45	Ischemic	52.04 ± 7.93/51.86 ± 7.81	ACT	Usual care	7w/7session/40-60 min	Nurses	Group/Face-to-face	Baseline, post-intervention	SDS, SAS, SCSQ, Treatment adherence, Patient satisfaction*
Yang (2023) (43)	45/45	Mixed	NR	ACT	Usual care	8w/16session/40 min	Psychological therapists and rehabilitation therapists	Individual/Face-to-face	Baseline, post-intervention	HAMD, HAMA, Hs-CRP
Liu (2022) (44)	32/33	Mixed	NR/ NR	ACT	Usual care	6w/7session/45-60 min	Multi-disciplinary team^2^	Individual and Group/Face-to-face and online	Baseline, post-intervention,1 m follow-up	PHQ-9, GAD-7
Dai (2020) (45)	55/54	NR	NR	ACT	Usual care	2w/5-6session/30-45 min	Nurses	Individual/Face-to-face	Baseline, post-intervention	AAQ-II, PTGI
Shi (2021) (25)	40/41	Ischemic	63.32 ± 1.17/62.51 ± 1.23	ACT	Usual care	4w/8session/20 min	Nurses	Individual/Face-to-face and online	Baseline, 2w, post-intervention	GAD-7, GDS-15, GSES
Zhou (2023) (46)	33/33	Mixed	67.7 ± 3.670/67.85 ± 4.848	ACT	Usual care	8w/8session/40–60 min	Nurses	Group/Face-to-face	Baseline, post-intervention, 1 m follow-up	HAMD-24, AAQ-II
Huang (2023) (47)	34/34	Ischemic	61.25 ± 3.53/62.38 ± 4.07	ACT	Usual care	4w/NR/NR	Neurologist and nurses	NR/Face-to-face and online	Baseline, post-intervention	GSES, SAS, SDS, BI, NIHSS, Complications
Li (2022) (48)	60/60	Hemorrhagic	61.22 ± 6.89/60.47 ± 7.13	ACT	Usual care	1w/4-7session/15–20 min	Nurses	Face-to-face	Baseline, post-intervention	GSES, Fugl-Meyer
Hao (2022) (49)	62/60	Hemorrhagic	43.5 ± 3.25/44.1 ± 4.16	ACT	Usual care	NR/NR/NR	Nurses	Face-to-face	Baseline, post-intervention	KPS, ADL, SWLS, SDS, SAS
Liu (2022) (50)	45/45	Hemorrhagic	59.22 ± 10.27/59.04 ± 10.35	ACT	Usual care	4w/8session/60 min	Nurses	NR	Baseline, post-intervention	MSSNS, BIPQ, GQOLI-74
Ding (2025) (51)	40/40	NR	66.22 ± 4.97/65.17 ± 5.05	ACT	Usual care	NR/NR/30 min	Psychological therapist and Nurses	Face-to-face	Baseline, post-intervention	Fugl-Meyer, FAM, FAC, NIHSS, HAMA, HAMD
Wang (2023) (20)	40/40	Hemorrhagic	64.12 ± 2.47/63.95 ± 2.51	ACT	Usual care	3w/9session/30 min	Nurses	Face-to-face	Baseline, post-intervention	FOP-Q-SF, SV-SS-QoL

NR, not reported; C, control; I, intervention; w, week; m, month; *, self-developed scale; ACT, Acceptance and Commitment Therapy; BI, Barthel Index; Mixed, Ischemic/Hemorrhagic; ^1^Multi-disciplinary team1: clinical psychologist per site, clinical psychologists, assistant psychologists, or stroke care co-ordinators; ^2^Multi-disciplinary team2: nurses, neurologist, brain-heart health manager, and psychological nursing expert.

### 3.3 Risk of bias

Inter-rater reliability between the two reviewers was substantial (*κ* = 0.697, z = 3.29, *p* < 0.001). Among the 22 included studies, 9 (40.9%) were classified as high risk of bias, and 13 (59.1%) as having some concerns. The risk of bias was primarily attributable to limitations in several areas. Common limitations included incomplete description of randomization, the absence of intention-to-treat analyses, the use of self-report scales, unclear trial registration information, and inadequate reporting of results. Detailed assessments are presented in Figure 2.

**Figure 2:**
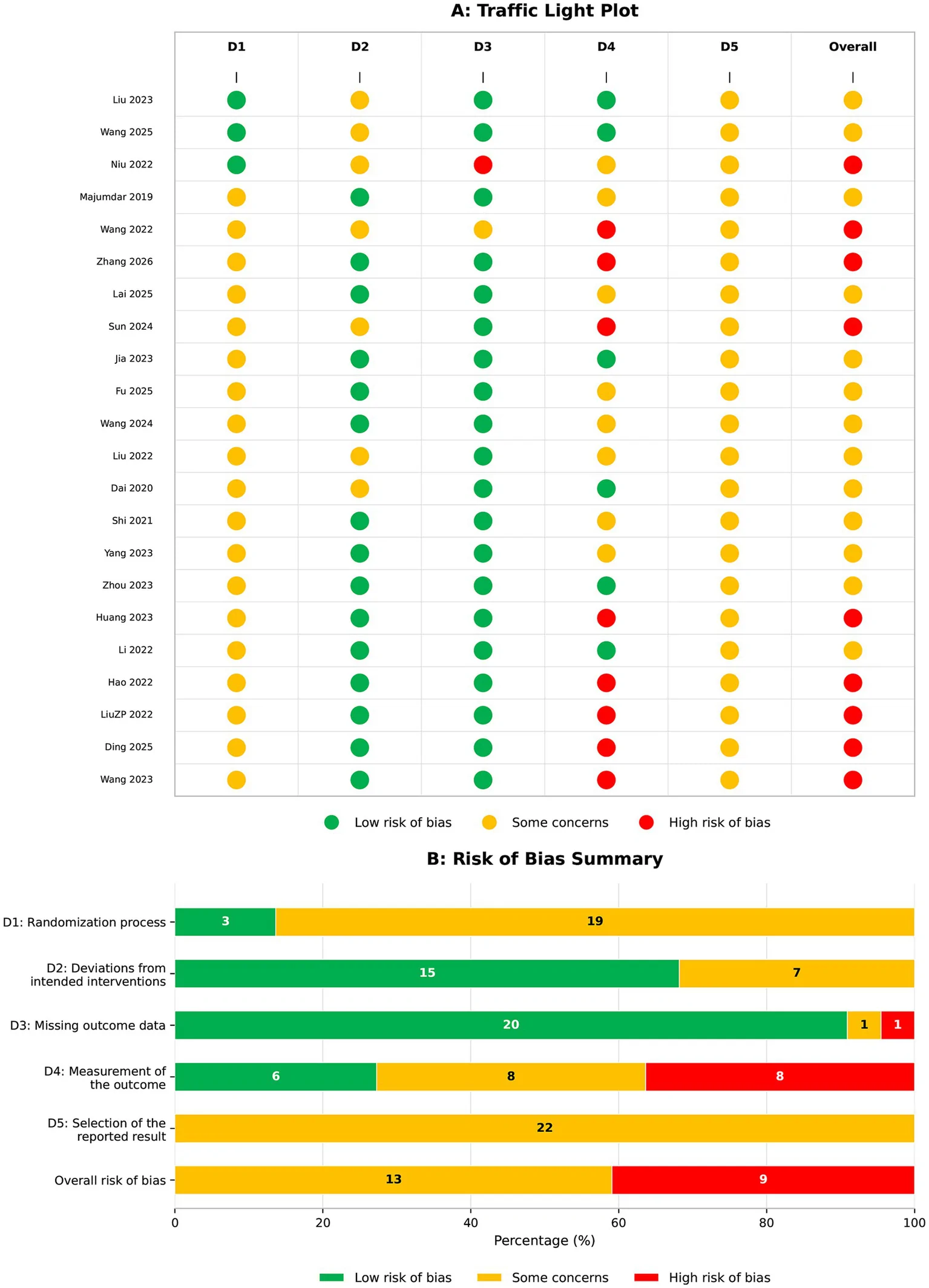
Risk of bias included studies. **(A)** Risk of bias graph. **(B)** Risk of bias summary. D1: Randomization process; D2: Deviations from intended interventions; D3: Missing outcome data; D4: Measurement of the outcome; D5: Selection of the reported result; Overall: Overall bias.

### 3.4 Outcomes

#### 3.4.1 Primary outcomes: depression and anxiety

Depression: Sixteen of the 22 included studies indicate that ACT may be associated with a positive effect on depressive symptoms in stroke patients in the post-intervention period [SMD = −1.32, 95% CI −1.80 to −0.84, *p* < 0.001, *I^2^* = 93.68%]. At short-term follow-up (≤3 months), 5 of the 22 included studies showed that the reduction remained statistically significant [SMD = −0.76, 95% CI −1.07 to −0.45, *p* < 0.001, *I^2^* = 53.82%]. The detailed content is shown in Figure 3.

**Figure 3:**
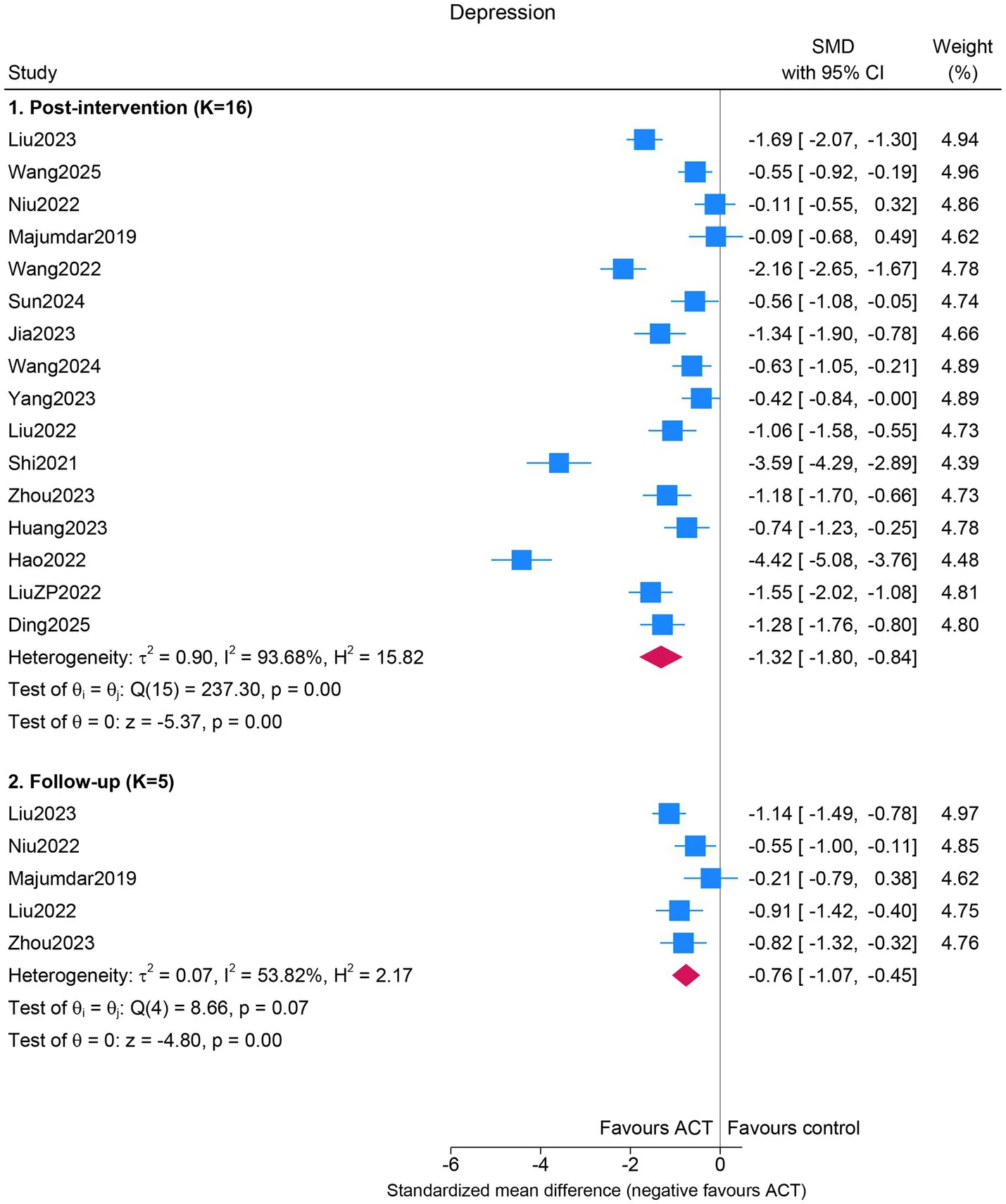
Forest plot of meta-analysis for the effect of ACT on depression. “*p* = 0.00” indicates a *p*-value below the software’s display threshold and should be interpreted as *p* < 0.001, consistent with the main text.

Anxiety: Thirteen of the 22 included studies, a reduction in anxiety levels was observed following the intervention [SMD = −1.36, 95% CI −1.90 to −0.82, *p* < 0.001, *I^2^* = 93.60%]. At short-term follow-up (≤3 months), two studies showed no significant between-group difference [SMD = −0.51, 95% CI −1.42 to 0.40, *p* = 0.276, *I^2^* = 81.79%]. The detailed content is shown in Figure 4.

**Figure 4:**
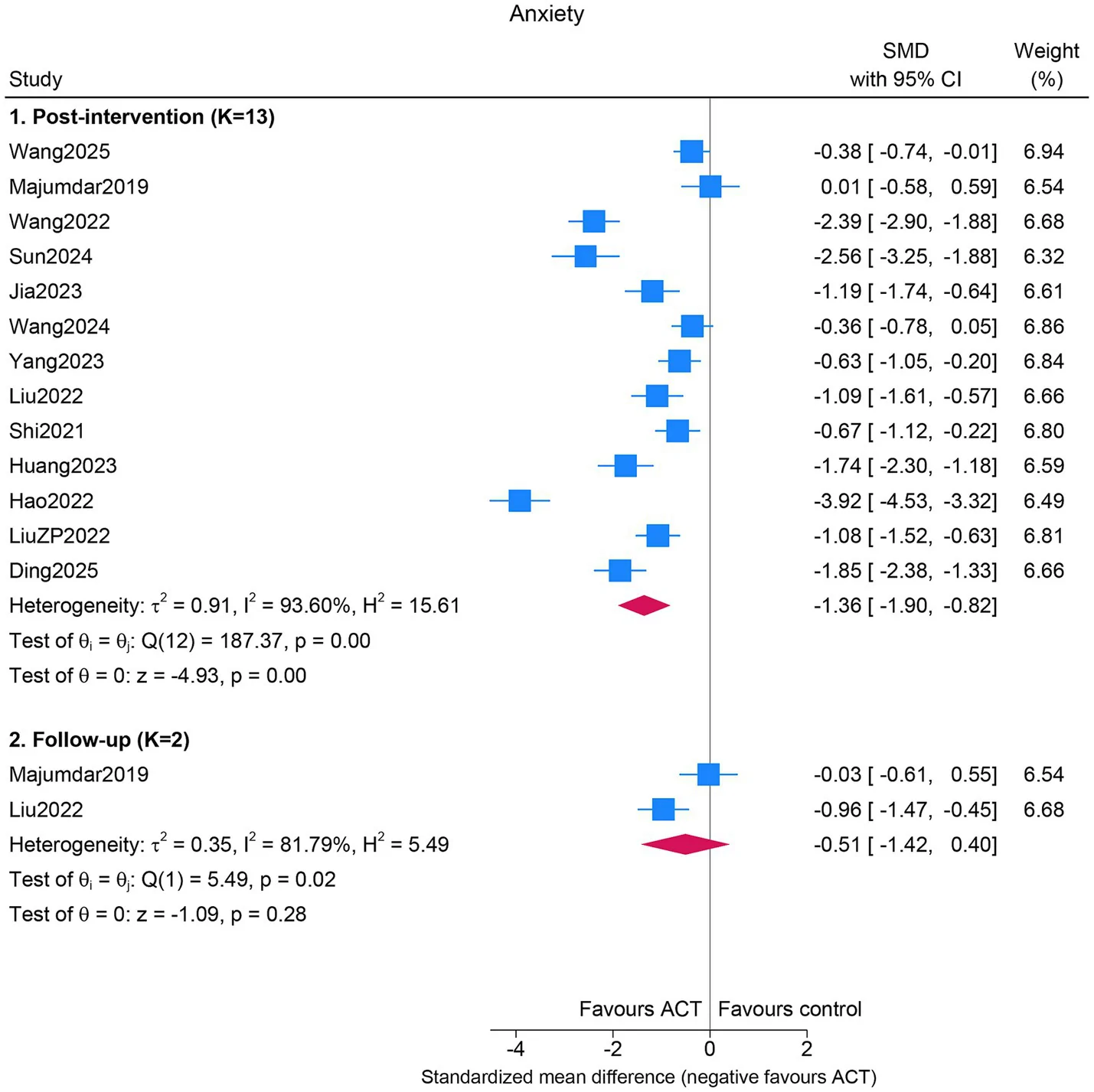
Forest plot of meta-analysis for the effect of ACT on anxiety. “*p* = 0.00” indicates a *p*-value below the software’s display threshold and should be interpreted as *p* < 0.001, consistent with the main text.

#### 3.4.2 Secondary outcomes

##### 3.4.2.1 Psychological processes

Psychological Flexibility: Seven of the 22 included studies reported post-intervention improvements [MD = −7.23, 95% CI −9.72 to −4.73, *p* < 0.001, *I^2^* = 91.36%], with effects remaining statistically significant in three studies at short-term follow-up (≤3 months) [MD = −4.79, 95% CI −5.65 to −3.93, *p* < 0.001, *I^2^* = 0.00%]. The detailed content is shown in Figure 5.

**Figure 5:**
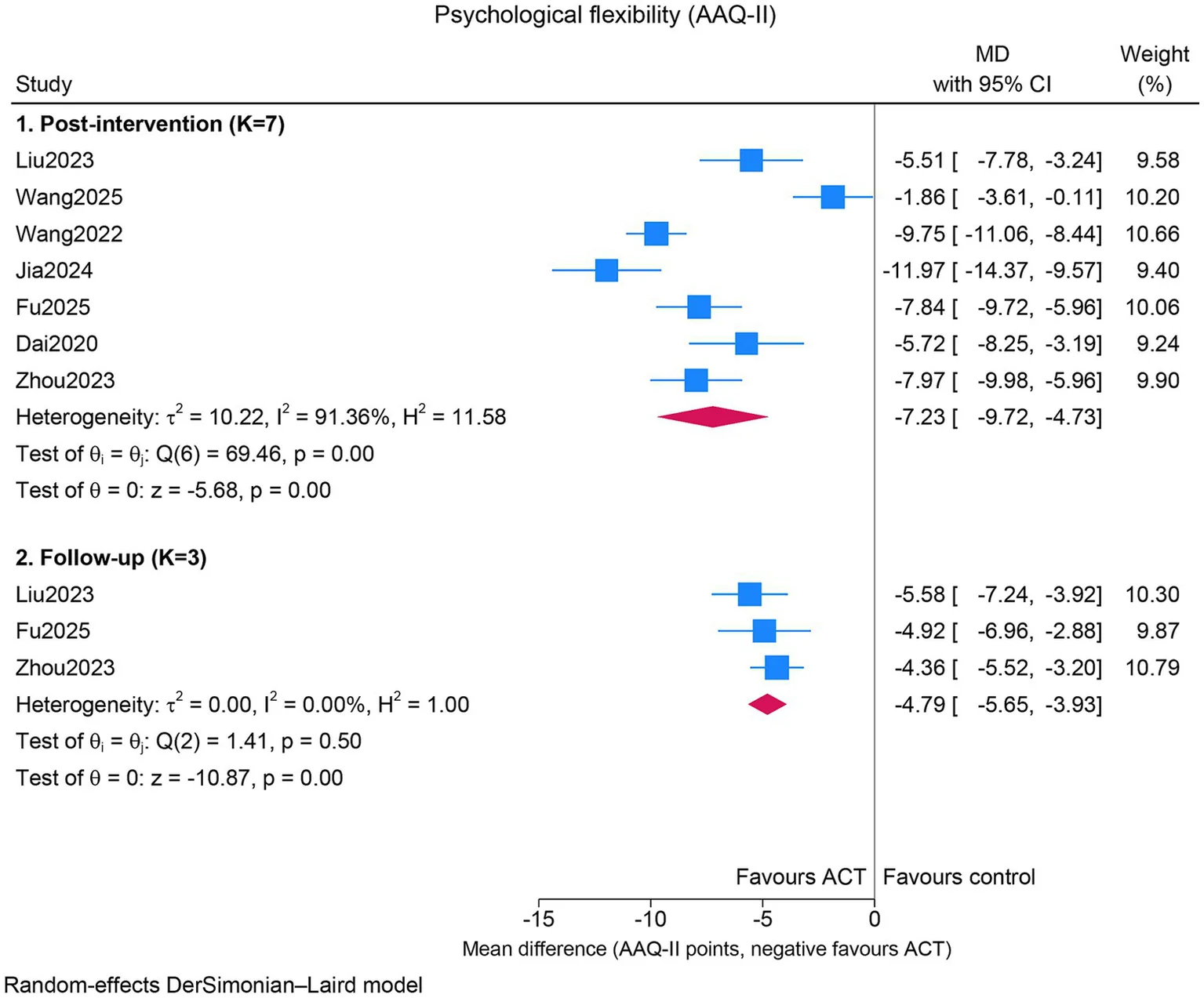
Forest plot of meta-analysis for the effect of ACT on psychological flexibility.

Cognitive Fusion: Four of the 22 included studies showed post-intervention reductions [SMD = −1.68, 95% CI −2.73 to −0.62, *p* = 0.002, *I^2^* = 95.52%]; follow-up in two studies suggested a maintained improvement at short-term follow-up [SMD = −1.26, 95% CI −2.10 to −0.42, *p* = 0.003, *I^2^* = 86.05%]. The detailed content is shown in Figure 6.

**Figure 6:**
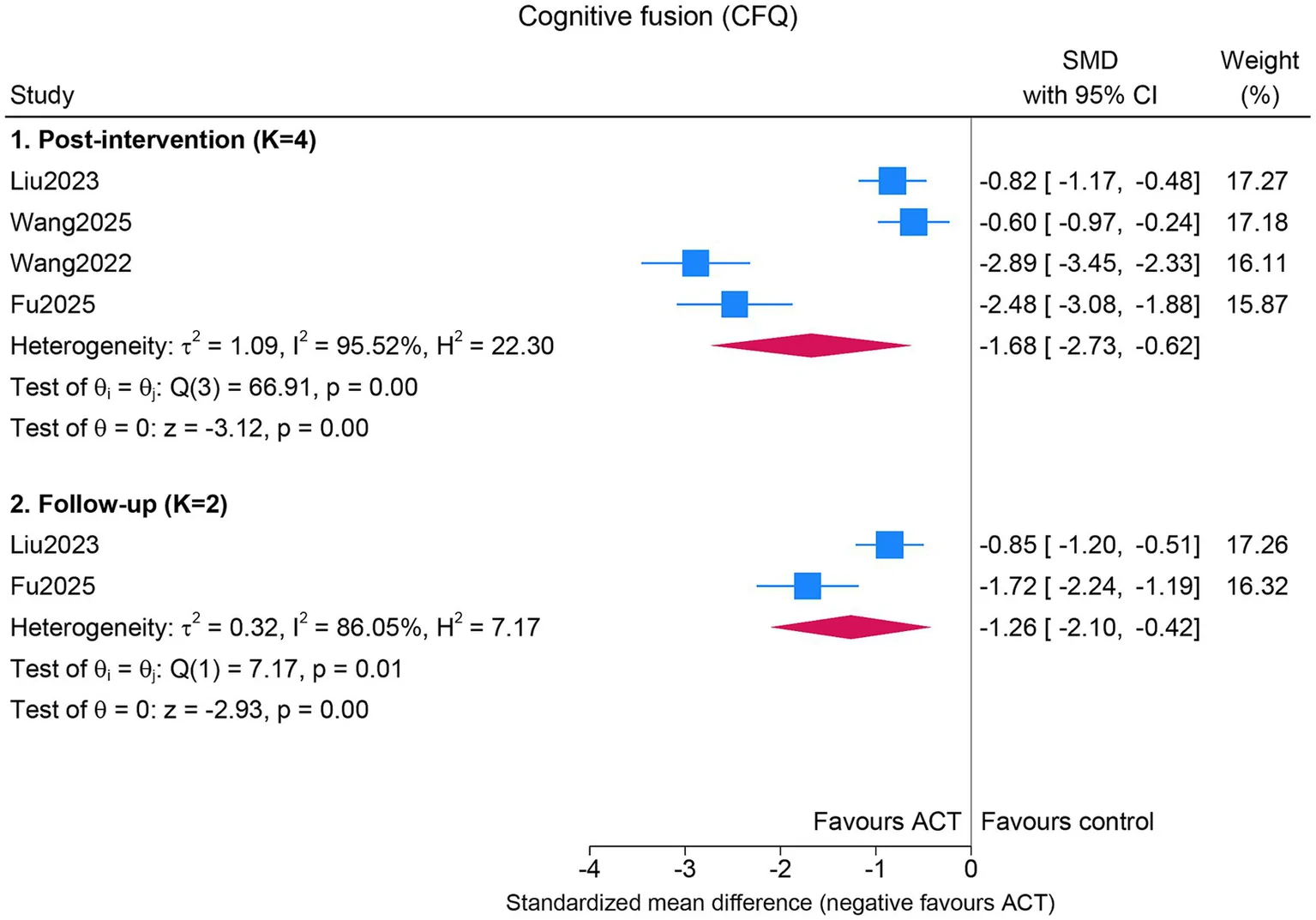
Forest plot of meta-analysis for the effect of ACT on cognitive fusion.

##### 3.4.2.2 Clinical outcomes

Quality of life: (1) For overall quality of life measured by total scores, three of the 22 included studies showed a significant improvement post-intervention [SMD = 1.21, 95% CI 0.03 to 2.39, *p* = 0.04, *I^2^* = 92.26%]. The detailed forest plots are shown in Figure 7.

**Figure 7:**
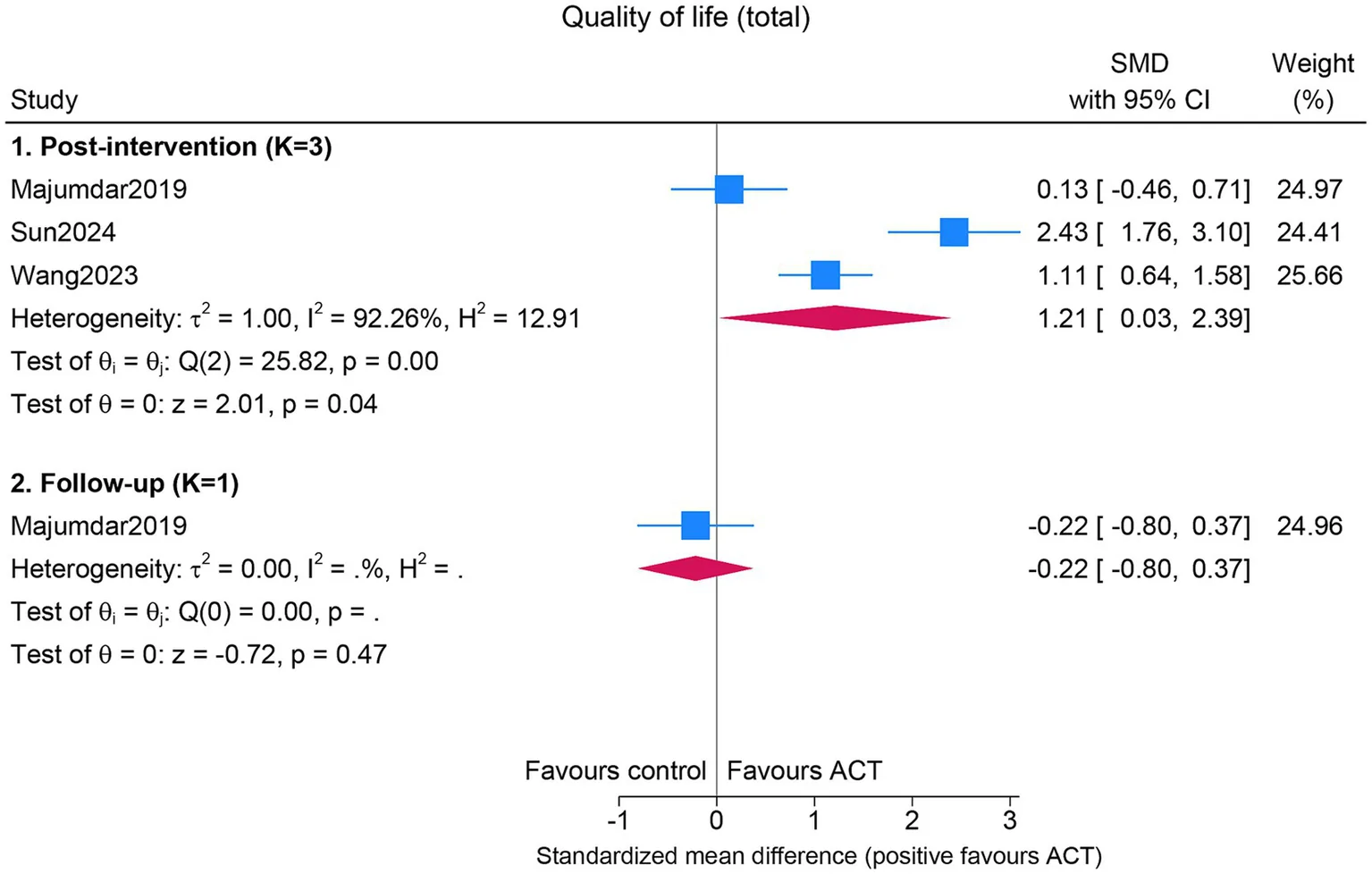
Forest plot of meta-analysis for the effect of ACT on quality of life.

(2) For the psychological domain of quality of life, three studies also yielded a significant effect favoring ACT [SMD = 1.21, 95% CI 0.61 to 1.81, *p* < 0.001, *I^2^* = 81.08%]; this estimate remained significant in all leave-one-out refits. The detailed forest plots are shown in Supplementary Figure S1.

Sleep quality: Four of the 22 included studies reported post-intervention improvement [MD = −2.50, 95% CI −3.41 to −1.60, *p* < 0.001, *I^2^* = 74.68%]. The detailed content is shown in Figure 8.

**Figure 8:**
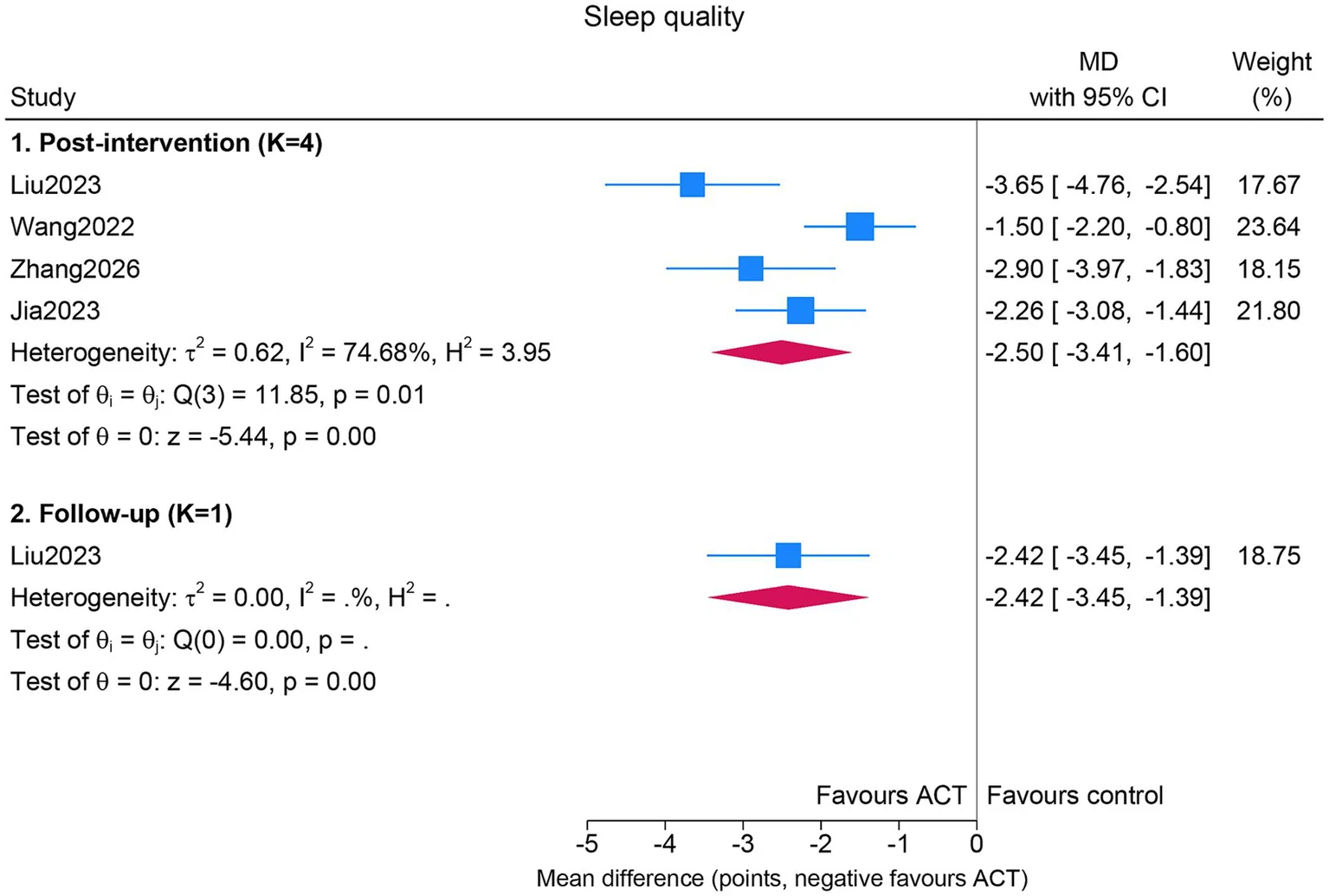
Forest plot of meta-analysis for the effect of ACT on sleep quality. “*p* = 0.00” indicates a *p*-value below the software’s display threshold and should be interpreted as *p* < 0.001, consistent with the main text.

Activities of daily living (ADL): Three of the 22 included studies showed that there was no significant effect at post-intervention [SMD = 1.08, 95% CI −0.66 to 2.81, *p* = 0.22, *I^2^* = 97.48%]. As shown in Figure 9.

**Figure 9:**
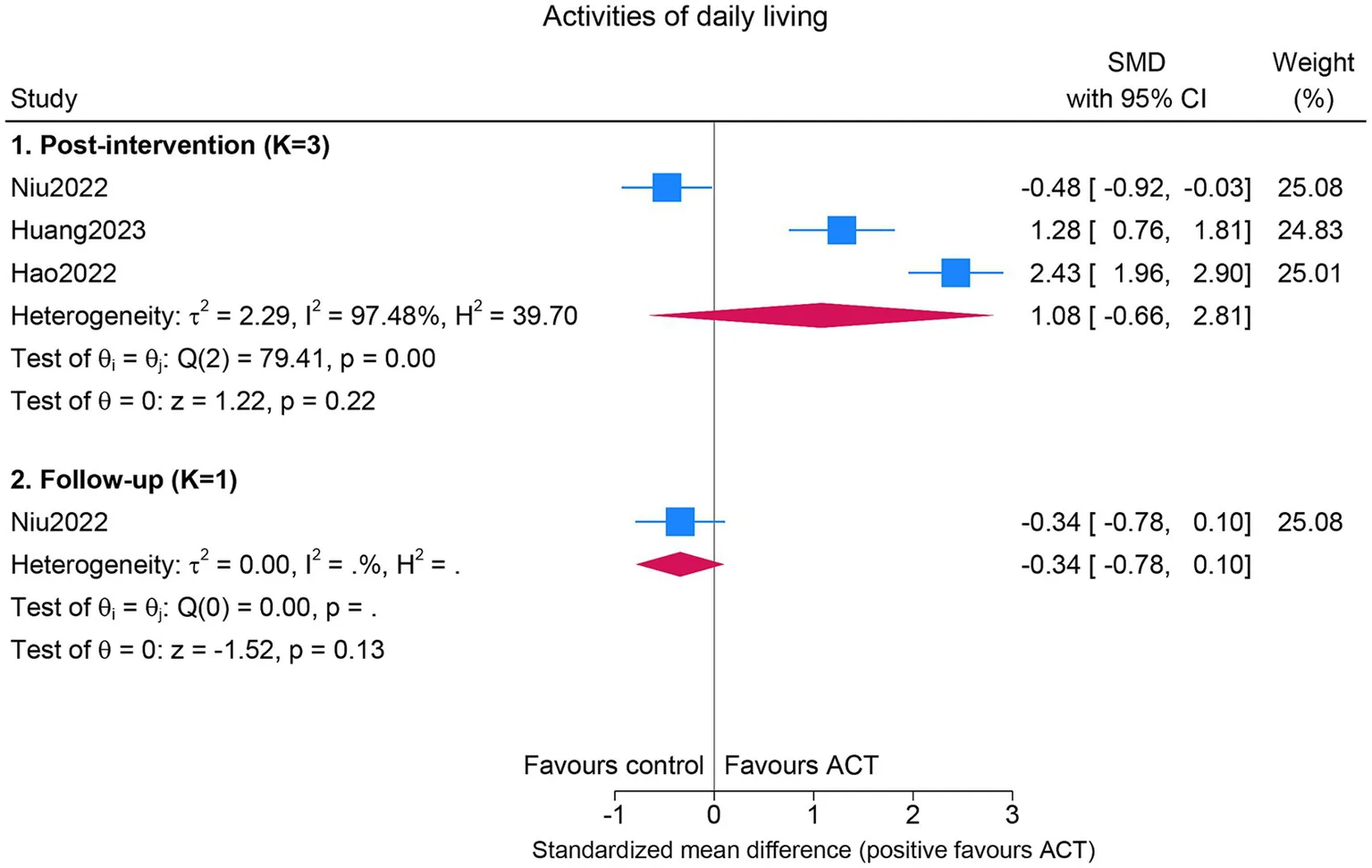
Forest plot of meta-analysis for the effect of ACT on ADL.

Neurological function: Three of the 22 included studies found non-significant post-intervention effects [MD = −2.79, 95% CI −5.89 to 0.31, *p* = 0.08, *I^2^* = 98.10%]. Specific details are shown in Figure 10.

**Figure 10:**
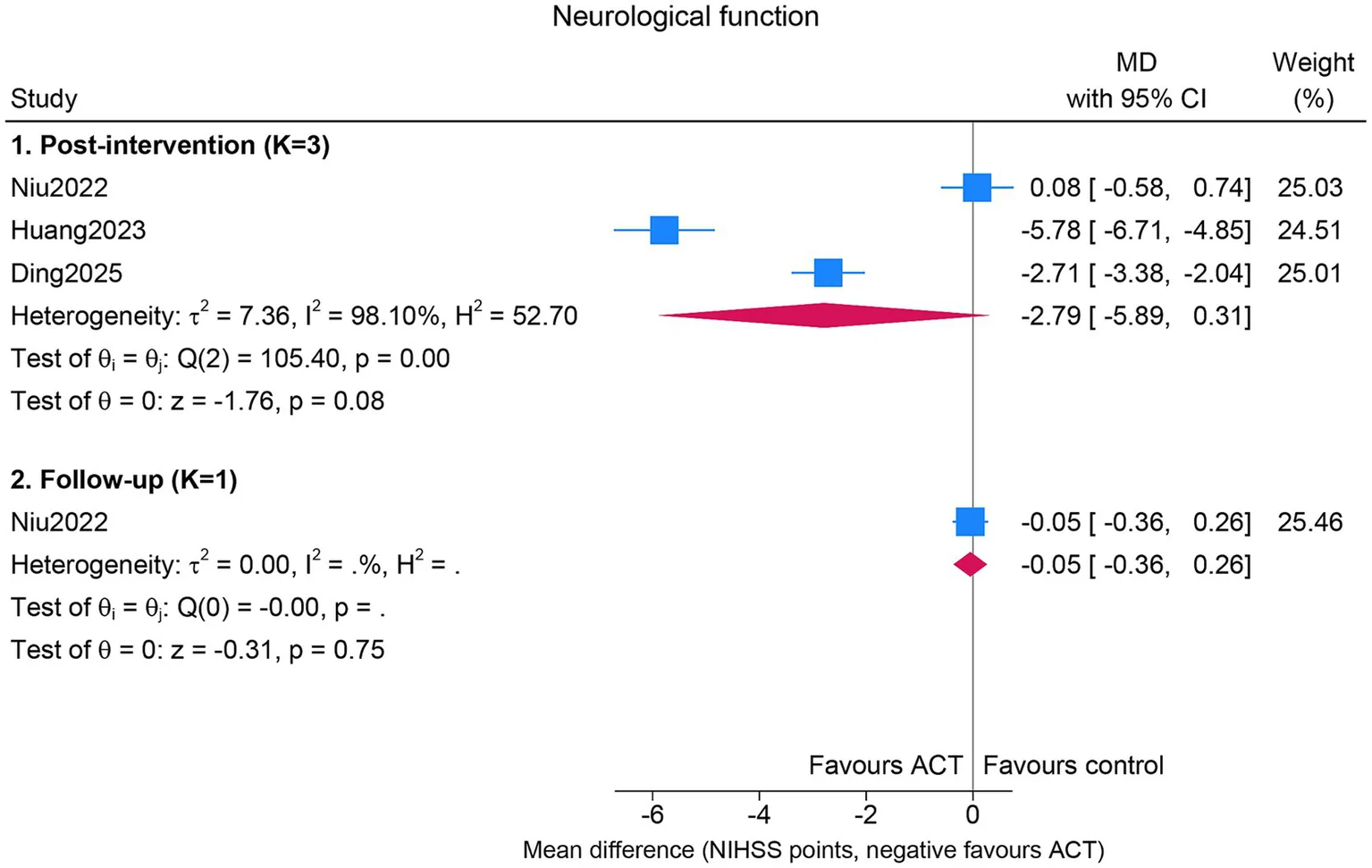
Forest plot of meta-analysis for the effect of ACT on neurological function.

##### 3.4.2.3 Positive psychological resources

Self-Efficacy: Four of the 22 included studies reported improvements at post-intervention [SMD = 1.02, 95% CI 0.68 to 1.36, *p* < 0.001, *I^2^* = 66.05%]; a follow-up study showed that the effect remained unchanged. The detailed content is shown in Figure 11.

**Figure 11:**
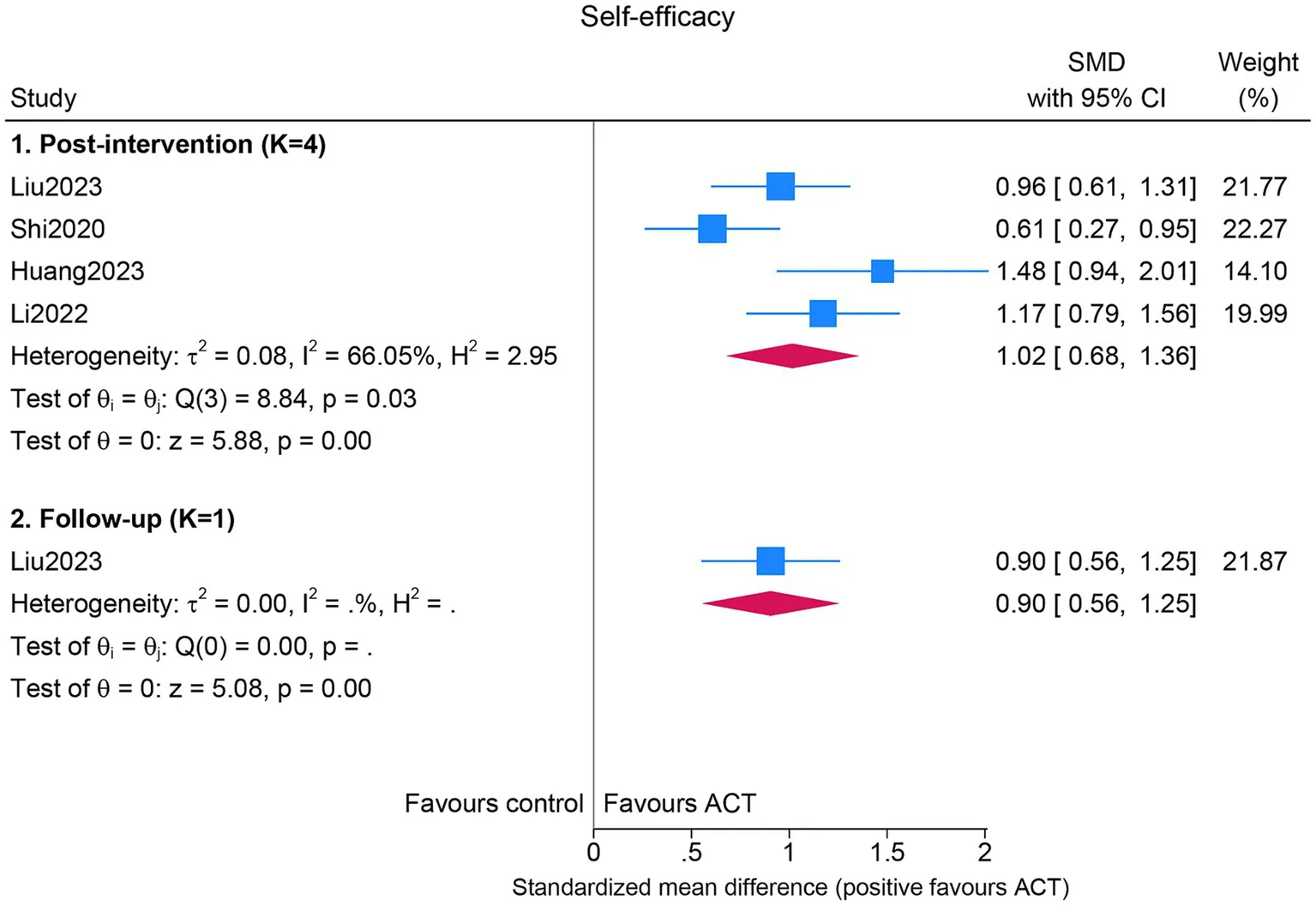
Forest plot of meta-analysis for the effect of ACT on self-efficacy. “*p* = 0.00” indicates a *p*-value below the software’s display threshold and should be interpreted as *p* < 0.001, consistent with the main text.

Hope: Two of the 22 included studies observed post-intervention improvement [SMD = 1.06, 95% CI -0.42 to 2.53, *p* = 0.16, *I^2^* = 94.11%]. The detailed content is shown in Supplementary Figure S2.

Resilience: Two of the 22 included studies reported improvement of psychological resilience at post-intervention [MD = 3.87, 95% CI 1.50 to 6.23, *p* = 0.001, *I*^2^ = 23.31%]; a later study (19) reported that the improvement remained statistically significant at short-term follow-up [MD = 2.16, 95% CI 0.62 to 3.70, *p* = 0.006]. Specific details are shown in Supplementary Figure S3.

#### 3.4.3 Attrition

A total of 16 studies reported dropout rates. Among the 16 studies reporting attrition, 12 reported zero events in both groups and 4 reported at least one event. All 16 studies were included in the pooled analysis, with zero-event studies handled using continuity correction [RR = 0.99, 95% CI 0.58 to 1.69, *p* =  0.96]. Dropouts occurred in 17 of 737 (2.3%) participants in the ACT groups and 17 of 735 (2.3%) in the control groups. There was no statistically significant difference in dropout rates between the ACT and control groups. Specific details are shown in Figure 12. There was no evidence of heterogeneity (*I^2^* = 0.00%, *p* = 1.00).

**Figure 12:**
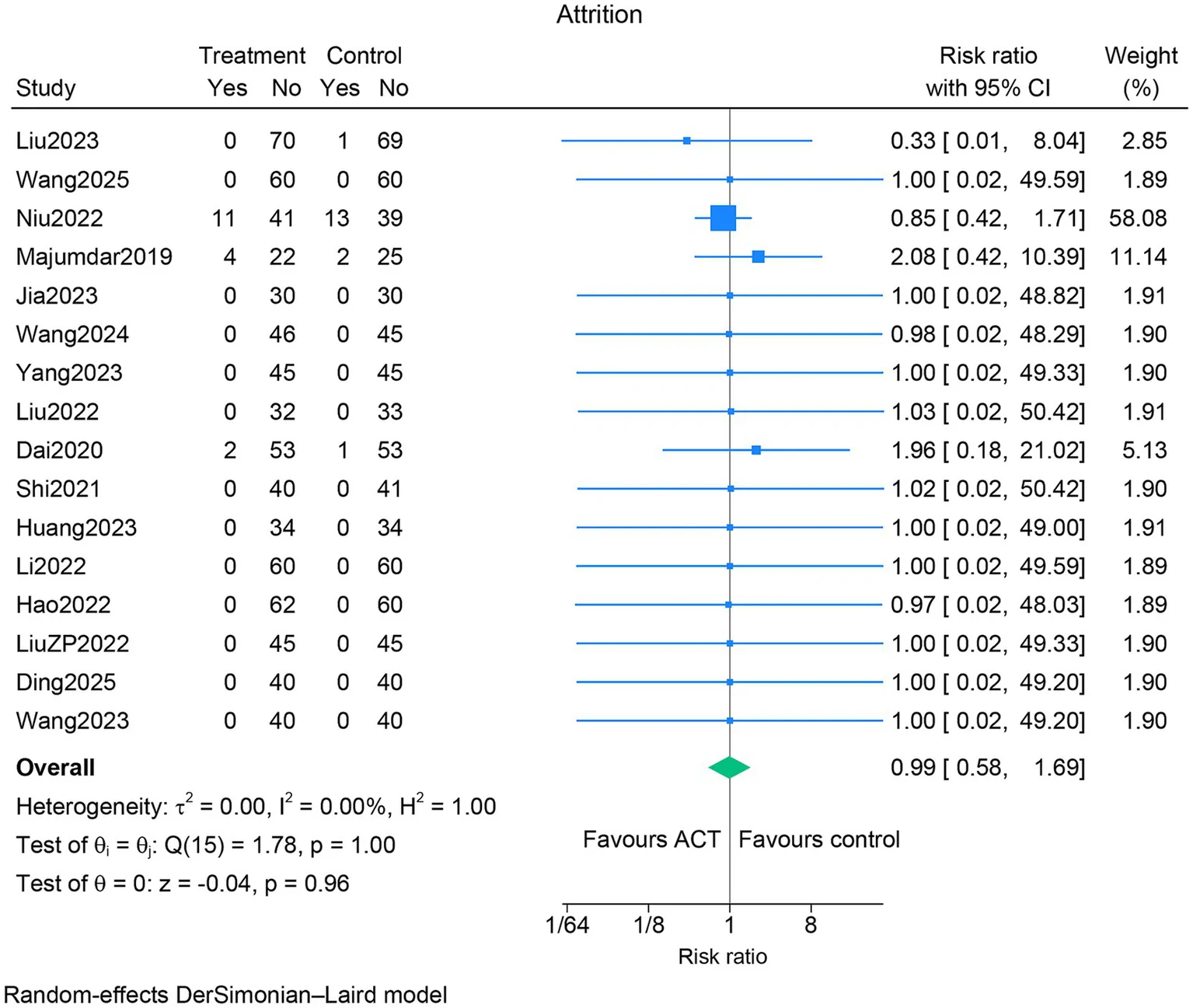
Forest plot of attrition outcomes of ACT in stroke patients.

### 3.5 Subgroup analyses

Detailed subgroup estimates are presented in Appendix 3: Supplementary Tables S1–S12. At post-intervention, no significant subgroup differences were observed for depression or for psychological flexibility. For anxiety, delivery format was significant (*p* = 0.005), with individual delivery showing a larger effect than group delivery. For quality of life, the total score differed across measurement scales (*p* = 0.025). For self-efficacy, the duration subgroup difference was statistically significant (*p* = 0.015), with short-duration interventions (≤4 weeks) showing a larger effect than longer-duration interventions (>4 weeks). ADL showed significant subgroup differences by stroke subtype and by measurement scale (both *p* = 0.026). All significant subgroup findings were based on small numbers of studies and should be regarded as exploratory.

At follow-up, subgroup analyses showed no significant differences across subgroups for depression or for psychological flexibility. For anxiety, quality of life, and cognitive fusion, the number of studies within strata was insufficient for any between-subgroup test. Given that some subgroups contained only a single study, these results should be interpreted descriptively rather than inferentially. Despite these subgroup analyses, within-stratum heterogeneity remained high for every subgrouping variable examined. Therefore, the pooled estimates represent average effects across studies and should be interpreted accordingly.

### 3.6 Meta-regression analysis

Meta-regression analyses were performed for depression, anxiety, and psychological flexibility to examine whether intervention duration was associated with effect size. No statistically significant association was found for these outcomes (all *p* > 0.05), and intervention duration explained little between-study variance (*R^2^* = 0.0–11.6%). These findings suggest that the observed heterogeneity is not explained by intervention duration. Because the number of studies was limited, and the AAQ-II analysis was exploratory and hypothesis-generating, these results should be interpreted with caution. Leave-one-out re-estimation suggested possible sensitivity to individual studies. The detailed results are presented in Supplementary Tables S13, S14.

### 3.7 Sensitivity analyses

Sensitivity analyses using a leave-one-out approach were performed to assess the robustness of the pooled estimates across post-intervention and follow-up outcomes. Details of the sensitivity analysis are presented in Appendix 5: Supplementary Figures S4–S15.

#### 3.7.1 Post-intervention outcomes

Sensitivity analysis showed that the pooled effects on anxiety, depression and psychological flexibility remained statistically significant and consistent regardless of which single study was omitted, whereas the total quality of life estimate (*K* = 3) was not robust: omitting Wang et al. (20) rendered the effect non-significant (SMD = 1.27, 95% CI −0.98 to 3.53). Although heterogeneity was reduced after excluding individual studies, substantial heterogeneity remained. Given the limited number of included studies, the results for neurological function and ADL were highly sensitive to the exclusion of individual studies, suggesting that these findings are fragile and should be interpreted with caution.

#### 3.7.2 Follow-up outcomes

Regarding the follow-up results, pooled effects for depression and psychological flexibility remained statistically significant, and excluding any one of the studies did not result in any substantial change (depression SMD range −0.88 to −0.64; psychological flexibility MD range −5.32 to −4.50). The pooled effect for anxiety was not statistically significant at follow-up (*K* = 2, *p* = 0.276).

### 3.8 Publication bias

Potential small-study effects were assessed using funnel plots and Egger’s test when at least 10 studies were available and the outcome was considered suitable for these analyses. In Egger’s test, a *p* value below 0.1 was considered indicative of small-study effects. Egger’s test suggested potential small-study effects for depression and anxiety (*p* = 0.025 and *p* = 0.012). Trim-and-fill analyses identified no potentially missing studies for either depression or anxiety, and the pooled effect estimates remained unchanged after adjustment (depression: SMD = −1.323, 95% CI: −1.896 to −0.750; anxiety: SMD = −1.360, 95% CI: −1.950 to −0.769). However, given the limited number of studies and the high heterogeneity, these analyses cannot exclude publication bias. For attrition, because zero-event studies were prevalent, formal assessment of small-study effects was not performed. Funnel plots for the eligible outcomes are presented in Appendix 6: Figures S16, S17.

### 3.9 GRADE assessment

The GRADE assessment indicated that the certainty of evidence ranged from very low to moderate across outcomes. The GRADE Evidence Profile and Summary of Findings tables are presented in Appendix 8. At post-intervention, the certainty of evidence was rated as low for dropout rates, depression, anxiety, and psychological flexibility. At follow-up, the certainty of evidence was rated as moderate for depression, low for psychological flexibility and very low for anxiety, mainly due to risk of bias, inconsistency, and imprecision. No serious concerns regarding indirectness were identified across outcomes. No outcome was downgraded for publication bias: although Egger’s tests suggested potential small-study effects for depression and anxiety, trim-and-fill analyses imputed no studies and the adjusted estimates remained unchanged.

## 4 Discussion

### 4.1 Principal findings

This systematic review and meta-analysis, including 22 randomized controlled trials (*n* = 1,975), evaluated the effects of Acceptance and Commitment Therapy (ACT) on psychological outcomes in stroke patients and explored the maintenance of treatment effects at short-term follow-up (≤3 months) for outcomes with available follow-up data. Attrition rates were also compared between the ACT and control groups.

Overall, our findings indicate that ACT may provide beneficial effects on psychological outcomes among stroke survivors. ACT may reduce depressive and anxiety symptoms at post-intervention. At short-term follow-up, the reduction in depressive symptoms remained statistically significant, whereas no significant between-group difference was observed for anxiety, suggesting that the durability of psychological effects may vary across outcomes and remains uncertain. In addition, ACT may be associated with improvements in several psychological processes and psychosocial outcomes, including psychological flexibility, cognitive fusion, quality of life, sleep quality, self-efficacy, and psychological resilience.

However, these findings were based on a limited number of studies, and heterogeneity was substantial for most outcomes. At short-term follow-up, improvements were observed in pooled analyses of psychological flexibility and cognitive fusion; these findings should therefore be regarded as preliminary and exploratory. These findings suggest that ACT may facilitate psychological adaptation to stroke-related challenges, potentially reflecting changes in maladaptive psychological processes and strengthening of positive psychological resources. However, evidence for effects on functional recovery was less consistent. No statistically significant benefits were observed for activities of daily living or neurological function, and these analyses were based on a small number of studies with substantial heterogeneity. Therefore, ACT may be better viewed as a psychological adjunct to conventional stroke rehabilitation, with the more apparent benefits occurring in psychological and psychosocial domains, whereas its potential to promote physical and functional recovery remains uncertain and warrants further investigation.

### 4.2 Comparison with previous reviews

A recent meta-analysis by Wang et al. (13) provided important evidence supporting the beneficial effects of ACT on psychological outcomes in stroke patients. Compared with this previous review, the present study additionally examined ACT-related psychological processes, explored treatment effects at follow-up for outcomes with available follow-up data, and assessed the certainty of evidence using GRADE. Although our findings were consistent with previous evidence regarding the immediate benefits of ACT, the attenuation of effects observed at short-term follow-up, most clearly for anxiety, suggests that the longer-term effects of ACT remain uncertain and warrant further investigation. In addition, the present review highlights that evidence for functional outcomes, such as activities of daily living and neurological function, remains limited and inconclusive, indicating a need for further well-powered studies in these domains.

### 4.3 Effects of ACT on depression and anxiety

#### 4.3.1 Depression

This meta-analysis suggested that ACT may be associated with reductions in depressive symptoms among stroke patients. The pooled estimate suggested a large effect at post-intervention, although heterogeneity was high; at short-term follow-up (≤3 months) the reduction remained statistically significant but was smaller. ACT may therefore provide immediate benefits that may be maintained at short-term follow-up for post-stroke depressive symptoms. The benefit may operate through enhanced psychological flexibility, acceptance, and value-based action, potentially interrupting the cycle between functional impairment and emotional distress (3, 11, 21). Subgroup analyses and meta-regression did not identify intervention duration, measurement instruments, stroke subtype, or delivery format as significant moderators; given the small number of studies in each stratum, these null findings should not be interpreted as evidence of no moderation. The attenuation after completion may reflect reduced therapist support; booster sessions or integration into routine rehabilitation may help maintain gains. Given the limited number of studies, these findings should be interpreted with caution.

#### 4.3.2 Anxiety

The ACT was associated with a moderate-to-large reduction in anxiety at post-intervention, but at short-term follow-up only two studies remained and the between-group effect was no longer statistically significant; immediate benefits may therefore not be maintained beyond the intervention period. The pooled post-intervention estimate should be read as an average tendency rather than a uniform benefit. A similar mechanism to that proposed for depression may be involved—enhanced psychological flexibility and reduced experiential avoidance (11)—particularly given that anxiety after stroke often reflects uncertainty about recovery, functional limitations, and fear of recurrence (5). Delivery format moderated post-intervention effects, with individual-based but not group-based delivery reaching significance; the small, imbalanced subgroups preclude any claim of superiority, and the format effect could not be assessed at short-term follow-up. This may reflect greater tailoring and therapist attention in individual sessions, whereas group delivery offers peer support and potential cost advantages. Duration was not a significant moderator, consistent with previous literature (17); however, the slope became significant only when Majumdar and Morris (22) was omitted, indicating sensitivity to a single study. Overall, high heterogeneity limits the certainty of these estimates, which should be regarded as provisional.

### 4.4 Psychological processes

#### 4.4.1 Psychological flexibility

This meta-analysis suggested that ACT may be associated with improvements in psychological flexibility: the pooled post-intervention estimate was significant, although heterogeneity was very high. Psychological inflexibility—experiential avoidance and cognitive fusion—may hinder rehabilitation engagement (23), and ACT may support psychological adaptation by strengthening acceptance and value-based action (7, 15). Subgroup analyses and meta-regression identified no significant moderators of duration, stroke subtype, or format, but stratum sizes were small (24); leave-one-out refits remained significant (Supplementary Figure S7), a stability that should nonetheless be viewed cautiously with only seven studies. At short-term follow-up the effect remained significant without heterogeneity, although robustness assessed from three studies is tentative.

#### 4.4.2 Cognitive fusion

The ACT may be associated with reduced cognitive fusion at post-intervention; cognitive fusion is central to psychological inflexibility and may mediate the relationship between psychological flexibility and post-stroke depression (11, 12), and defusion techniques may help patients distance themselves from maladaptive thoughts (11). The duration subgroup analysis showed no difference between short-duration (≤4w) and long-duration (>4w) interventions. However, both subgroups showed considerable heterogeneity, indicating that other unmeasured factors, such as differences in intervention content, participant characteristics, or study implementation, may contribute to variability among studies. Therefore, these subgroup findings should be interpreted cautiously, and further large-sample studies are needed for validation. For post-intervention cognitive fusion (*K* = 4), leave-one-out refits remained significant, but heterogeneity persisted. However, only two studies reported follow-up data for cognitive fusion (*K* = 2); therefore, a leave-one-out sensitivity analysis was not performed for the follow-up time point, as the pooled estimate would be driven entirely by a single study.

### 4.5 Rehabilitation-related and functional outcomes

#### 4.5.1 Quality of life

The ACT may be associated with better quality of life, but each analysis included only three studies, so the findings are exploratory. Leave-one-out refits remained significant for the psychological domain but not for total quality of life when Wang (2023) was omitted; the instrument subgroup difference (*p* = 0.025) rested on a single non-SS-QOL study, so the influence of measurement scales remains uncertain.

#### 4.5.2 Sleep

The ACT may be associated with improvements in sleep quality immediately after intervention. Leave-one-out analyses showed that the pooled estimate remained statistically significant and that no single study materially changed the overall effect, suggesting that the pooled estimate was not driven by any individual study. However, heterogeneity remained substantial, and the absence of a significant moderator effect for duration may reflect limited statistical power and a restricted range of intervention durations; therefore, a duration effect cannot be excluded.

#### 4.5.3 Self-efficacy and resilience

The ACT may be associated with improvements in self-efficacy at post-intervention; leave-one-out refits stayed significant, with Shi and Wang (25) accounting for part of the heterogeneity, and acceptance and value-guided action may support more consistent behavioral engagement (26, 27). For resilience, two studies suggested improvement, although the evidence is very limited; resilience, defined as maintaining adaptive responses under stress (28, 29), may be enhanced through psychological flexibility processes (11).

#### 4.5.4 Hope and functional outcomes

Evidence is insufficient to support clear benefits for hope, activities of daily living, or neurological function. Hope, an important correlate of rehabilitation motivation and adaptation (30), showed no significant between-group difference, with a wide confidence interval indicating uncertainty in the effect estimate. Activities of daily living and neurological function likewise showed no significant between-group effects and appeared sensitive to individual studies. Notably, the effect on neurological function became statistically significant only after excluding Niu et al. (31) (MD = −4.23, 95% CI − 7.24 to −1.22), suggesting that this finding was not robust and should therefore be interpreted cautiously. Because these domains are shaped mainly by neurological impairment and rehabilitation intensity (32, 33), ACT is best regarded as a psychological adjunct, and future studies should use larger samples and consistent follow-up assessment.

### 4.6 Attrition and clinical implications

#### 4.6.1 Attrition: dropout rates

This meta-analysis found no statistically significant difference in attrition between the ACT and control groups, with relatively low attrition rates in both groups, the risk difference was essentially zero. These findings suggest that ACT was not associated with a higher risk of participant attrition than the control condition, and that participant retention was broadly comparable between the two groups. Attrition reflects whether participants remain in a study until the end of the observation period and is commonly reported in psychotherapy and behavioral intervention trials as an indicator of participant retention (34). However, all-cause attrition may arise from a range of factors unrelated to the intervention itself, including adverse events, changes in medical condition, hospitalization, transfer to another facility, logistical barriers, loss to follow-up, caregiver circumstances, or other unrelated events (24, 34). Therefore, the comparable attrition observed in this meta-analysis was interpreted primarily as evidence of similar participant retention between groups. Stroke patients may commonly experience functional impairment, cognitive deficits, and dependence on caregivers, which may create additional challenges for intervention participation and participant retention in psychological intervention trials (10, 32). The included studies showed no substantial statistical heterogeneity in attrition. However, of the 16 studies included, 12 reported zero attrition events in both arms and only four reported any events, which contributed to the pooled risk ratio; the risk difference was estimated using all 16 studies. Given this distribution of zero-event studies, formal assessment of small-study effects was not performed for this outcome. In addition, attrition data were not reported for seven of the 22 included studies, which may introduce some uncertainty into the pooled estimate. Overall, the available evidence suggests that ACT was not associated with increased participant attrition compared with control conditions among stroke survivors. However, attrition alone does not identify the reasons for dropout or directly capture participants’ experiences with the intervention. Future studies should report attrition together with specific reasons for dropout and use direct measures of treatment acceptability, adherence, and engagement to provide a more comprehensive evaluation of ACT in different stroke populations and intervention formats.

#### 4.6.2 Clinical implications

These findings suggest that ACT may serve as a useful adjunct to conventional stroke rehabilitation, particularly for improving psychological distress and maladaptive psychological processes such as psychological inflexibility and cognitive fusion. The intervention was not associated with higher dropout rates in the available studies; however, all-cause attrition alone cannot establish feasibility, acceptability, or adherence, and these constructs require direct measurement in future trials. However, because evidence for functional outcomes remains limited and follow-up data are sparse, ACT should currently be viewed as a complementary rather than substitutive approach. Future studies should clarify which delivery formats and patient subgroups derive the greatest benefit, and whether booster sessions or continued support are needed to maintain gains over time.

### 4.7 Limitations and future directions

Several limitations should be considered when interpreting these findings. Although only randomized controlled trials were included, the overall methodological quality remained suboptimal. Most studies were rated as having some concerns, and a minority were judged to be at high risk of bias, mainly due to inadequate reporting of randomization, absence of intention-to-treat analyses, and reliance on self-reported outcomes.

Substantial heterogeneity was observed across major outcomes, and predefined subgroup and meta-regression analyses did not fully explain this variability. This may be attributable to unmeasured factors, such as differences in patient characteristics (e.g., stroke severity and cognitive function) and therapist expertise. Future studies should therefore clearly specify patients’ baseline characteristics at enrollment (e.g., stroke severity, cognitive function, time since stroke, baseline symptom severity, and comorbid conditions), which would enable stratified analyses, help clarify sources of between-study heterogeneity, and identify which patients are most likely to benefit from ACT. Some trials did not report key dose components (number of sessions, session frequency, or session duration), which may limit the assessment of dose–response relationships. The evidence base for several outcomes was also limited, with few studies and sparse follow-up data. Given the high heterogeneity across these outcomes, the certainty of evidence is limited and these findings should be considered preliminary.

Generalizability may be constrained by the predominance of studies conducted in China and variations in intervention formats. Egger’s test suggested potential small-study effects for depression (*p* = 0.025) and anxiety (*p* = 0.012). However, because the number of studies was limited and between-study heterogeneity was very high, Egger’s test had low power and trim-and-fill estimates were unreliable; publication bias therefore cannot be excluded. Finally, most outcomes were assessed only at short-term follow-up (≤3 months after the end of the intervention), no long-term (>3 months) data were available, and the long-term sustainability of ACT remains uncertain. Follow-up evidence was sparse and unevenly distributed: pooled short-term estimates were available only for depression, anxiety, psychological flexibility, and cognitive fusion, whereas each of the remaining outcomes had at most one usable follow-up study or none at all, precluding pooled analysis; their durability therefore remains unknown and should be examined in future studies with consistent follow-up time points.

## 5 Conclusion

This meta-analysis suggests that ACT may be associated with improvements in depression, anxiety, and psychological flexibility in stroke patients, with benefits remaining at short-term follow-up for depression and psychological flexibility, though not for anxiety. ACT may also be associated with improvements in related psychological processes, and did not appear to increase dropout rates in the available studies. However, evidence for functional outcomes and hope remains limited. These findings suggest that ACT may be a useful adjunct to conventional stroke rehabilitation, particularly for addressing psychological distress. Because the evidence remains preliminary, further high-quality trials with larger samples and longer follow-up are needed to confirm its long-term effectiveness and optimal implementation strategies.

## Data Availability

The original contributions presented in the study are included in the article/Supplementary material, further inquiries can be directed to the corresponding author.
