# Supplementary material for: Effects of acceptance and commitment therapy on psychological and health-related outcomes in stroke patients: a systematic review and meta-analysis of randomized controlled trials
Source: Front Neurol. 2026 Oct 28;17:1953111. doi: 10.3389/fneur.2026.1953111 (PMC13751444; doi:10.3389/fneur.2026.1953111)
Supplement: Supplementary file 1 [file Supplementary_file_1.docx]

**Appendix 1. PRISMA 2020 Checklist**

| **Section and Topic** | **Item #** | **Checklist item** | **Location where item is reported** |
| --- | --- | --- | --- |
| **TITLE** | | |  |
| Title | 1 | Identify the report as a systematic review. |  |
| **ABSTRACT** | | |  |
| Abstract | 2 | See the PRISMA 2020 for Abstracts checklist. |  |
| **INTRODUCTION** | | |  |
| Rationale | 3 | Describe the rationale for the review in the context of existing knowledge. |  |
| Objectives | 4 | Provide an explicit statement of the objective(s) or question(s) the review addresses. |  |
| **METHODS** | | |  |
| Eligibility criteria | 5 | Specify the inclusion and exclusion criteria for the review and how studies were grouped for the syntheses. |  |
| Information sources | 6 | Specify all databases, registers, websites, organisations, reference lists and other sources searched or consulted to identify studies. Specify the date when each source was last searched or consulted. |  |
| Search strategy | 7 | Present the full search strategies for all databases, registers and websites, including any filters and limits used. |  |
| Selection process | 8 | Specify the methods used to decide whether a study met the inclusion criteria of the review, including how many reviewers screened each record and each report retrieved, whether they worked independently, and if applicable, details of automation tools used in the process. |  |
| Data collection process | 9 | Specify the methods used to collect data from reports, including how many reviewers collected data from each report, whether they worked independently, any processes for obtaining or confirming data from study investigators, and if applicable, details of automation tools used in the process. |  |
| Data items | 10a | List and define all outcomes for which data were sought. Specify whether all results that were compatible with each outcome domain in each study were sought (e.g. for all measures, time points, analyses), and if not, the methods used to decide which results to collect. |  |
|  | 10b | List and define all other variables for which data were sought (e.g. participant and intervention characteristics, funding sources). Describe any assumptions made about any missing or unclear information. |  |
| Study risk of bias assessment | 11 | Specify the methods used to assess risk of bias in the included studies, including details of the tool(s) used, how many reviewers assessed each study and whether they worked independently, and if applicable, details of automation tools used in the process. |  |
| Effect measures | 12 | Specify for each outcome the effect measure(s) (e.g. risk ratio, mean difference) used in the synthesis or presentation of results. |  |
| Synthesis methods | 13a | Describe the processes used to decide which studies were eligible for each synthesis (e.g. tabulating the study intervention characteristics and comparing against the planned groups for each synthesis (item #5)). |  |
|  | 13b | Describe any methods required to prepare the data for presentation or synthesis, such as handling of missing summary statistics, or data conversions. |  |
|  | 13c | Describe any methods used to tabulate or visually display results of individual studies and syntheses. |  |
|  | 13d | Describe any methods used to synthesize results and provide a rationale for the choice(s). If meta-analysis was performed, describe the model(s), method(s) to identify the presence and extent of statistical heterogeneity, and software package(s) used. |  |
|  | 13e | Describe any methods used to explore possible causes of heterogeneity among study results (e.g. subgroup analysis, meta-regression). |  |
|  | 13f | Describe any sensitivity analyses conducted to assess robustness of the synthesized results. |  |
| Reporting bias assessment | 14 | Describe any methods used to assess risk of bias due to missing results in a synthesis (arising from reporting biases). |  |
| Certainty assessment | 15 | Describe any methods used to assess certainty (or confidence) in the body of evidence for an outcome. |  |
| **RESULTS** | | |  |
| Study selection | 16a | Describe the results of the search and selection process, from the number of records identified in the search to the number of studies included in the review, ideally using a flow diagram. |  |
|  | 16b | Cite studies that might appear to meet the inclusion criteria, but which were excluded, and explain why they were excluded. |  |
| Study characteristics | 17 | Cite each included study and present its characteristics. |  |
| Risk of bias in studies | 18 | Present assessments of risk of bias for each included study. |  |
| Results of individual studies | 19 | For all outcomes, present, for each study: (a) summary statistics for each group (where appropriate) and (b) an effect estimate and its precision (e.g. confidence/credible interval), ideally using structured tables or plots. |  |
| Results of syntheses | 20a | For each synthesis, briefly summarise the characteristics and risk of bias among contributing studies. |  |
|  | 20b | Present results of all statistical syntheses conducted. If meta-analysis was done, present for each the summary estimate and its precision (e.g. confidence/credible interval) and measures of statistical heterogeneity. If comparing groups, describe the direction of the effect. |  |
|  | 20c | Present results of all investigations of possible causes of heterogeneity among study results. |  |
|  | 20d | Present results of all sensitivity analyses conducted to assess the robustness of the synthesized results. |  |
| Reporting biases | 21 | Present assessments of risk of bias due to missing results (arising from reporting biases) for each synthesis assessed. |  |
| Certainty of evidence | 22 | Present assessments of certainty (or confidence) in the body of evidence for each outcome assessed. |  |
| **DISCUSSION** | | |  |
| Discussion | 23a | Provide a general interpretation of the results in the context of other evidence. |  |
|  | 23b | Discuss any limitations of the evidence included in the review. |  |
|  | 23c | Discuss any limitations of the review processes used. |  |
|  | 23d | Discuss implications of the results for practice, policy, and future research. |  |
| **OTHER INFORMATION** | | |  |
| Registration and protocol | 24a | Provide registration information for the review, including register name and registration number, or state that the review was not registered. |  |
|  | 24b | Indicate where the review protocol can be accessed, or state that a protocol was not prepared. |  |
|  | 24c | Describe and explain any amendments to information provided at registration or in the protocol. |  |
| Support | 25 | Describe sources of financial or non-financial support for the review, and the role of the funders or sponsors in the review. |  |
| Competing interests | 26 | Declare any competing interests of review authors. |  |
| Availability of data, code and other materials | 27 | Report which of the following are publicly available and where they can be found: template data collection forms; data extracted from included studies; data used for all analyses; analytic code; any other materials used in the review. |  |

*From:*  Page MJ, McKenzie JE, Bossuyt PM, Boutron I, Hoffmann TC, Mulrow CD, et al. The PRISMA 2020 statement: an updated guideline for reporting systematic reviews. BMJ 2021;372:n71. doi: 10.1136/bmj.n71. This work is licensed under CC BY 4.0. To view a copy of this license, visit <https://creativecommons.org/licenses/by/4.0/>

**Appendix 2. Search strategy**

| **PubMed** | |
| --- | --- |
| #1 | ("Stroke"[Mesh]) AND "Acceptance and Commitment Therapy"[Mesh] |
| #2 | ((Stroke[Title/Abstract] OR Apoplexy[Title/Abstract] OR CVA[Title/Abstract] OR "Cerebrovascular Accident*"[Title/Abstract] OR "Vascular Accident"[Title/Abstract]) OR "Cerebral Hemorrhage"[Title/Abstract]) AND ("Acceptance and Commitment Therapy"[Title/Abstract]) |
| #3 | #1 OR #2 |

| **Embase** | |
| --- | --- |
| #1 | 'cerebrovascular accident'/exp |
| #2 | 'accident, cerebrovascular':ti,ab,kw OR 'acute cerebrovascular lesion':ti,ab,kw OR 'acute focal cerebral vasculopathy':ti,ab,kw OR 'acute stroke':ti,ab,kw OR 'apoplectic stroke':ti,ab,kw OR 'apoplexia':ti,ab,kw OR 'apoplexy':ti,ab,kw OR 'blood flow disturbance, brain':ti,ab,kw OR 'brain accident':ti,ab,kw OR 'brain attack':ti,ab,kw OR 'brain blood flow disturbance':ti,ab,kw OR 'brain insult':ti,ab,kw OR 'brain insultus':ti,ab,kw OR 'brain vascular accident':ti,ab,kw OR 'cerebral apoplexia':ti,ab,kw OR 'cerebral insult':ti,ab,kw OR 'cerebral stroke':ti,ab,kw OR 'cerebral vascular accident':ti,ab,kw OR 'cerebral vascular insufficiency':ti,ab,kw OR 'cerebro vascular accident':ti,ab,kw OR 'cerebrovascular arrest':ti,ab,kw OR 'cerebrovascular failure':ti,ab,kw OR 'cerebrovascular injury':ti,ab,kw OR 'cerebrovascular insufficiency':ti,ab,kw OR 'cerebrovascular insult':ti,ab,kw OR 'cerebrum vascular accident':ti,ab,kw OR 'cryptogenic stroke':ti,ab,kw OR 'cva':ti,ab,kw OR 'insultus cerebralis':ti,ab,kw OR 'ischaemic seizure':ti,ab,kw OR 'ischemic seizure':ti,ab,kw OR 'stroke':ti,ab,kw OR 'thrombotic stroke':ti,ab,kw OR 'cerebrovascular accident':ti,ab,kw |
| #3 | #1 OR #2 |
| #4 | 'acceptance and commitment therapy'/exp |
| #5 | ('acceptance commitment therapy':ti,ab,kw OR 'act psychotherapy':ti,ab,kw OR acceptance:ti,ab,kw) AND 'commitment therapy':ti,ab,kw |
| #6 | #4 OR #5 |
| #7 | #3 AND #6 |

| **Web of Science** | |
| --- | --- |
| #1 | "Stroke" OR "Apoplexy" OR "CVA" OR "Cerebrovascular Accident*" OR "Vascular Accident" (Topic) |
| #2 | "Acceptance and Commitment Therapy" (Topic) |
| #3 | #1 AND #2 |

| **Medline** | |
| --- | --- |
| #1 | (Acceptance and Commitment Therapy) OR AB (Acceptance and Commitment Therapy) OR TI (Acceptance and Commitment Therapy) |
| #2 | (Stroke) OR AB ('Stroke' OR 'Apoplexy' OR 'CVA' OR 'Cerebrovascular Accident*' OR 'Vascular Accident' OR 'Cerebral Hemorrhage') OR TI ('Stroke' OR 'Apoplexy' OR 'CVA' OR 'Cerebrovascular Accident*' OR 'Vascular Accident' OR 'Cerebral Hemorrhage') |
| #3 | #1 AND #2 |

| **CINAHL** | |
| --- | --- |
| #1 | (Acceptance and Commitment Therapy) OR AB (Acceptance and Commitment Therapy) OR TI (Acceptance and Commitment Therapy) |
| #2 | (Stroke) OR AB ('Stroke' OR 'Apoplexy' OR 'CVA' OR 'Cerebrovascular Accident*' OR 'Vascular Accident' OR 'Cerebral Hemorrhage') OR TI ('Stroke' OR 'Apoplexy' OR 'CVA' OR 'Cerebrovascular Accident*' OR 'Vascular Accident' OR 'Cerebral Hemorrhage') |
| #3 | #1 AND #2 |

| **Cochrane Library** | |
| --- | --- |
| #1 | MeSH descriptor: [Acceptance and Commitment Therapy] explode all trees |
| #2 | MeSH descriptor: [Stroke] explode all trees |
| #3 | #1 AND #2 |
| #4 | (Stroke OR Apoplexy OR CVA OR (Cerebrovascular NEXT Accident*) OR (Vascular NEXT Accident)):ti,ab,kw |
| #5 | (Acceptance and Commitment Therapy):ti,ab,kw |
| #6 | #4 AND #5 |
| #7 | #3 OR #6 |

| **Scopus** | |
| --- | --- |
| #1 | TITLE-ABS-KEY ( Stroke OR Apoplexy OR CVA OR "Cerebrovascular Accident*" OR "Vascular Accident" ) |
| #2 | TITLE-ABS-KEY ( Acceptance AND Commitment Therapy ) |
| #3 | #1 AND #2 |

| **PsycInfo** | |
| --- | --- |
| #1 | TI ("Stroke" or "Apoplexy" or "CVA" or "Cerebrovascular Accident*" or "Vascular Accident") OR AB ("Stroke" or "Apoplexy" or "CVA" or "Cerebrovascular Accident*" or "Vascular Accident") |
| #2 | TI ("Acceptance and Commitment Therapy") OR AB ("Acceptance and Commitment Therapy") |
| #3 | #1 AND #2 |
| **CNKI** | |
| #1 | （主题：卒中 + 急性卒中 + 脑卒中 + 脑中风 + 脑血管意外 + CVA + 脑血管中风 +  急性脑血管意外 + 急性脑卒中 + 脑出血)  *Note:* (Topic: Stroke or Acute Stroke or Stroke or Cerebrovascular Accident or CVA or Cerebrovascular Stroke or Acute Cerebrovascular Accident or Acute Stroke or Cerebral Hemorrhage) |
| #2 | （主题：接纳承诺疗法 + 接纳承诺）  *Note:* (Topic: Acceptance and Commitment Therapy or Acceptance and Commitment) |
| #3 | #1 AND #2 |

| **VIP** | |
| --- | --- |
| #1 | 主题 = (卒中 or 急性卒中 or 脑卒中 or 脑中风 or 脑血管意外 or CVA or 脑血管中风 or  急性脑血管意外 or 急性脑卒中 or 脑出血)  *Note:* (Topic: Stroke or Acute Stroke or Stroke or Cerebrovascular Accident or CVA or Cerebrovascular Stroke or Acute Cerebrovascular Accident or Acute Stroke or Cerebral Hemorrhage) |
| #2 | 主题 = (接纳承诺疗法 or 接纳承诺）  *Note:* (Topic: Acceptance and Commitment Therapy or Acceptance and Commitment) |
| #3 | #1 AND #2 |

| **Wanfang** | |
| --- | --- |
| #1 | （主题：卒中 or 急性卒中 or 脑卒中 or 脑中风 or 脑血管意外 or CVA or 脑血管中风 or  急性脑血管意外 or 急性脑卒中or 脑出血)  *Note:* (Topic: Stroke or Acute Stroke or Stroke or Cerebrovascular Accident or CVA or Cerebrovascular Stroke or Acute Cerebrovascular Accident or Acute Stroke or Cerebral Hemorrhage) |
| #2 | （主题：接纳承诺疗法 or 接纳承诺）  *Note:* (Topic: Acceptance and Commitment Therapy or Acceptance and Commitment) |
| #3 | #1 AND #2 |

| **Sinomed** | |
| --- | --- |
| #1 | 主题:(卒中 or 急性卒中 or 脑卒中 or 脑中风 or 脑血管意外 or CVA or 脑血管中风 or  急性脑血管意外 or 急性脑卒中or 脑出血)  *Note:* (Topic: Stroke or Acute Stroke or Stroke or Cerebrovascular Accident or CVA or Cerebrovascular Stroke or Acute Cerebrovascular Accident or Acute Stroke or Cerebral Hemorrhage) |
| #2 | 主题:( 接纳承诺疗法 or 接纳承诺)  *Note:* Topic:(Acceptance and Commitment Therapy or Acceptance and Commitment) |
| #3 | #1 AND #2 |

| **Journal of Contextual Behavioral Science** | |
| --- | --- |
| #1 | Search term: 'Acceptance and Commitment' and 'stroke' |

**Appendix 3. Subgroup analyses and meta-regression analysis**

| **Table S1. Summary of subgroup analysis for depression at post-intervention.** | | | | | | | |
| --- | --- | --- | --- | --- | --- | --- | --- |
| **Subgroup analysis** | **K** | **SMD/MD [95% CI]** | **P** | **I² (%)** | **Q (df)** | **P (heterogeneity)** | **Subgroup difference** |
| **Overall effect** | 16 | -1.317 (-1.798, -0.836) | <0.001 | 93.7 | 237.30 (15) | <0.001 | — |
| **Duration** |  |  |  |  |  |  | Q = 0.47, df = 1, P = 0.492 |
| long (>4w) | 5 | -1.368 (-2.214, -0.521) | 0.002 | 93.5 | 61.16 (4) | <0.001 | — |
| short (≤4w) | 8 | -1.016 (-1.555, -0.477) | <0.001 | 90.4 | 73.05 (7) | <0.001 | — |
| **Stroke type** |  |  |  |  |  |  | Q = 3.68, df = 2, P = 0.158 |
| hemorrhagic | 2 | -2.976 (-5.792, -0.159) | 0.038 | 97.9 | 48.39 (1) | <0.001 | — |
| ischemic | 6 | -1.406 (-2.289, -0.523) | 0.002 | 94.6 | 92.60 (5) | <0.001 | — |
| mixed | 4 | -0.789 (-1.162, -0.416) | <0.001 | 56.6 | 6.91 (3) | 0.075 | — |
| **Delivery format** |  |  |  |  |  |  | Q = 3.41, df = 2, P = 0.182 |
| group | 5 | -0.751 (-1.385, -0.118) | 0.020 | 89.4 | 37.66 (4) | <0.001 | — |
| individual | 5 | -1.608 (-2.483, -0.732) | <0.001 | 93.2 | 58.68 (4) | <0.001 | — |
| others | 2 | -2.737 (-6.028, 0.553) | 0.103 | 98.4 | 61.54 (1) | <0.001 | — |
| **Scale** |  |  |  |  |  |  | Q = 1.50, df = 1, P = 0.221 |
| Clinician-rated scales | 5 | -0.936 (-1.538, -0.334) | 0.002 | 89.1 | 36.56 (4) | <0.001 | — |
| Self-reported scales | 11 | -1.500 (-2.174, -0.827) | <0.001 | 94.9 | 194.96 (10) | <0.001 | — |
| Note: K = number of studies; Q = Cochran's Q test for heterogeneity. P for subgroup difference was derived from the Q test between subgroups. | | | | | | | |

| **Table S2. Summary of subgroup analysis for anxiety at post-intervention.** | | | | | | | |
| --- | --- | --- | --- | --- | --- | --- | --- |
| **Subgroup analysis** | **K** | **SMD/MD [95% CI]** | **P** | **I² (%)** | **Q (df)** | **P (heterogeneity)** | **Subgroup difference** |
| **Overall effect** | 13 | -1.357 (-1.896, -0.818) | <0.001 | 93.6 | 187.37 (12) | <0.001 | — |
| **Duration** |  |  |  |  |  |  | Q = 2.60, df = 1, P = 0.107 |
| long (>4w) | 4 | -0.588 (-0.893, -0.282) | <0.001 | 50.3 | 6.03 (3) | 0.110 | — |
| short (≤4w) | 6 | -1.169 (-1.806, -0.532) | <0.001 | 89.5 | 47.78 (5) | <0.001 | — |
| **Stroke type** |  |  |  |  |  |  | Q = 0.71, df = 2, P = 0.702 |
| hemorrhagic | 2 | -2.490 (-5.280, 0.299) | 0.080 | 98.2 | 55.24 (1) | <0.001 | — |
| ischemic | 5 | -1.259 (-1.995, -0.523) | 0.001 | 91.1 | 44.99 (4) | <0.001 | — |
| mixed | 3 | -1.396 (-2.425, -0.367) | 0.008 | 91.1 | 22.36 (2) | <0.001 | — |
| **Delivery format** |  |  |  |  |  |  | Q = 10.70, df = 2, P = 0.005 |
| group | 2 | -0.237 (-0.583, 0.110) | 0.180 | 3.9 | 1.04 (1) | 0.308 | — |
| individual | 5 | -1.065 (-1.486, -0.644) | <0.001 | 74.7 | 15.80 (4) | 0.003 | — |
| others | 2 | -2.500 (-5.278, 0.277) | 0.078 | 97.9 | 48.24 (1) | <0.001 | — |
| **Scale** |  |  |  |  |  |  | Q = 0.05, df = 1, P = 0.825 |
| Clinician-rated scales | 2 | -1.230 (-2.430, -0.030) | 0.044 | 92.1 | 12.70 (1) | <0.001 | — |
| Self-reported scales | 11 | -1.382 (-2.009, -0.756) | <0.001 | 94.3 | 174.61 (10) | <0.001 | — |
| Note: K = number of studies; Q = Cochran's Q test for heterogeneity. P for subgroup difference was derived from the Q test between subgroups. | | | | | | | |

| **Table S3. Summary of subgroup analysis for psychological flexibility at post-intervention.** | | | | | | | |
| --- | --- | --- | --- | --- | --- | --- | --- |
| **Subgroup analysis** | **K** | **SMD/MD [95% CI]** | **P** | **I² (%)** | **Q (df)** | **P (heterogeneity)** | **Subgroup difference** |
| **Overall effect** | 7 | -7.227 (-9.719, -4.734) | <0.001 | 91.4 | 69.46 (6) | <0.001 | — |
| **Duration** |  |  |  |  |  |  | Q = 0.91, df = 1, P = 0.339 |
| long (>4w) | 3 | -5.870 (-9.949, -1.792) | 0.005 | 92.9 | 28.25 (2) | <0.001 | — |
| short (≤4w) | 4 | -8.287 (-11.108, -5.466) | <0.001 | 86.6 | 22.46 (3) | <0.001 | — |
| Stroke type |  |  |  |  |  |  | Q = 1.49, df = 1, P = 0.222 |
| ischemic | 3 | -9.737 (-11.733, -7.741) | <0.001 | 72.1 | 7.16 (2) | 0.028 | — |
| mixed | 1 | -7.970 (-9.984, -5.956) | <0.001 | — | — | — | — |
| **Delivery format** |  |  |  |  |  |  | Q = 0.26, df = 1, P = 0.608 |
| group | 3 | -7.208 (-8.685, -5.732) | <0.001 | 36.0 | 3.13 (2) | 0.209 | — |
| individual | 2 | -8.858 (-14.983, -2.733) | 0.005 | 91.9 | 12.32 (1) | <0.001 | — |
| Note: K = number of studies; Q = Cochran's Q test for heterogeneity; I² = proportion of variability attributable to heterogeneity. P values for each subgroup refer to the test of the pooled effect within that subgroup; the P value in the 'Subgroup difference' column refers to the Q test between subgroups. Pooling used a random-effects (DerSimonian–Laird) model. K for a given stratifier is lower than the overall K when studies with missing data for that variable were excluded; K values therefore do not sum to the overall K. | | | | | | | |

| **Table S4. Summary of subgroup analysis for cognitive fusion at post-intervention.** | | | | | | | |
| --- | --- | --- | --- | --- | --- | --- | --- |
| **Subgroup analysis** | **Effect Size** | |  | **Heterogeneity** | | | **Subgroup difference** |
|  | **K** | **SMD/MD [95% CI]** | ***P*** | **I^2^ (%)** | **Q** | ***P*** |  |
| **Overall effect** | 4 | -1.677 (-2.729, -0.624) | 0.002 | 95.5 | 66.91 (3) | <0.001 | — |
| **Duration** |  |  |  |  |  |  | Q = 0.05, df = 1, P = 0.820 |
| short (≤4w) | 2 | -1.526 (-3.366, 0.314) | 0.104 | 96.4 | 27.55 (1) | <0.001 |  |
| long (>4w) | 2 | -1.844 (-3.867, 0.178) | 0.074 | 97.3 | 37.71 (1) | <0.001 |  |
| Note: K = number of studies; Q = Cochran's Q test for heterogeneity. P for subgroup difference was derived from the Q test between subgroups. | | | | | | | |

| **Table S5. Summary of subgroup analysis for overall quality of life at post-intervention.** | | | | | | | |
| --- | --- | --- | --- | --- | --- | --- | --- |
| **Subgroup analysis** | **K** | **SMD/MD [95% CI]** | **P** | **I² (%)** | **Q (df)** | **P (heterogeneity)** | **Subgroup difference** |
| **Overall effect** | 3 | 1.212 (0.030, 2.395) | 0.044 | 92.3 | 25.82 (2) | <0.001 | — |
| **Duration** |  |  |  |  |  |  | — |
| short (≤4w) | 2 | 0.636 (-0.328, 1.601) | 0.196 | — | 6.60 (1) | 0.010 | — |
| **Scale** |  |  |  |  |  |  | Q = 5.04, df = 1, P = 0.025 |
| EQ5D | 1 | 0.128 (-0.457, 0.713) | 0.668 | — | — | — | — |
| SSQOL | 2 | 1.748 (0.460, 3.037) | 0.008 | 90.0 | 9.97 (1) | 0.002 | — |
| Note: K = number of studies; Q = Cochran's Q test for heterogeneity. P for subgroup difference was derived from the Q test between subgroups. | | | | | | | |

| **Table S6. Summary of subgroup analysis for QoL psychological dimension at post-intervention.** | | | | | | | |
| --- | --- | --- | --- | --- | --- | --- | --- |
| **Subgroup analysis** | **K** | **SMD/MD [95% CI]** | **P** | **I² (%)** | **Q (df)** | **P (heterogeneity)** | **Subgroup difference** |
| **Delivery format** |  |  |  |  |  |  | Q = 0.39, df = 1, P = 0.532 |
| group | 1 | 1.180 (0.820, 1.540) | <0.001 | — | — | — | — |
| individual | 2 | 1.260 (0.064, 2.455) | 0.039 | 90.4 | 10.39 (1) | 0.001 | — |
| Note: K = number of studies; Q = Cochran's Q test for heterogeneity. P for subgroup difference was derived from the Q test between subgroups. | | | | | | | |

| **Table S7. Summary of subgroup analysis for sleep at post-intervention.** | | | | | | | |
| --- | --- | --- | --- | --- | --- | --- | --- |
| **Subgroup analysis** | **K** | **SMD/MD [95% CI]** | **P** | **I² (%)** | **Q (df)** | **P (heterogeneity)** | **Subgroup difference** |
| **Overall effect** | 4 | -2.505(-3.408,-1.602) | <0.001 | 74.7 | 11.85 (3) | 0.008 | — |
| **Duration** |  |  |  |  |  |  | Q = 0.39, df = 1, P = 0.532 |
| long (>4w) | 1 | -2.900(-3.974,-1.826) | <0.001 | — | — | — | — |
| short (≤4w) | 3 | -2.402(-3.538,-1.265) | <0.001 | 80.7 | 10.36 (2) | 0.006 | — |
| Note: K = number of studies; Q = Cochran's Q test for heterogeneity. P for subgroup difference was derived from the Q test between subgroups. | | | | | | | |

| **Table S8. Summary of subgroup analysis for self-efficacy at post-intervention.** | | | | | | | |  |
| --- | --- | --- | --- | --- | --- | --- | --- | --- |
| **Subgroup analysis** | **K** | **SMD/MD [95% CI]** | **P** | **I² (%)** | **Q (df)** | **P (heterogeneity)** | **Subgroup difference** |  |
| **Overall effect** | 4 | 1.017 (0.678, 1.356) | <0.001 | 66.0 | 8.84 (3) | 0.032 | — |  |
| **Duration** |  |  |  |  |  |  | Q = 5.91, df = 1, P = 0.015 |  |
| long (>4w) | 1 | 0.607 (0.266, 0.948) | <0.001 | — | — | — | — |  |
| short (≤4w) | 3 | 1.147 (0.877, 1.418) | <0.001 | 23.0 | 2.60 (2) | 0.273 | — |  |
| **Stroke type** |  |  |  |  |  |  | Q = 0.11, df = 1, P = 0.743 |  |
| hemorrhagic | 1 | 1.172 (0.785, 1.560) | <0.001 | — | — | — | — |  |
| ischemic | 2 | 1.016 (0.165, 1.867) | 0.019 | 86.1 | 7.20 (1) | 0.007 | — |  |
| **Delivery format** |  |  |  |  |  |  | Q = 0.05, df = 1, P = 0.825 |  |
| group | 1 | 0.956 (0.605, 1.307) | <0.001 | — | — | — | — |  |
| individual | 2 | 0.882 (0.328, 1.436) | 0.002 | 78.3 | 4.61 (1) | 0.032 | — |  |
| **Scale** |  |  |  |  |  |  | Q = 0.10, df = 1, P = 0.749 |  |
| CaSM | 1 | 0.956 (0.605, 1.307) | <0.001 | — | — | — | — |  |
| GSES | 3 | 1.056 (0.553, 1.559) | <0.001 | 77.4 | 8.83 (2) | 0.012 | — |  |
| Note: K = number of studies; Q = Cochran's Q test for heterogeneity. P for subgroup difference was derived from the Q test between subgroups. | | | | | | | | |

| **Table S9. Summary of subgroup analysis for neurological function at post-intervention.** | | | | | | | |
| --- | --- | --- | --- | --- | --- | --- | --- |
| **Subgroup analysis** | **K** | **SMD/MD [95% CI]** | **P** | **I² (%)** | **Q (df)** | **P (heterogeneity)** | **Subgroup difference** |
| **Overall effect** | 3 | -2.789(-5.891, 0.313) | 0.078 | 98.1 | 105.40 (2) | <0.001 | — |
| **Delivery format** |  |  |  |  |  |  | — |
| group † | 1 | 0.080 (-0.580, 0.740) | 0.812 | — | — | — | — |
| individual † | 1 | -2.710(-3.379,-2.041) | <0.001 | — | — | — | — |
| **Scale** |  |  |  |  |  |  | Q = 0.00, df = 1, P = 0.965 |
| FMA | 1 | -2.710(-3.379,-2.041) | <0.001 | — | — | — | — |
| NIHSS | 2 | -2.841(-8.583, 2.902) | 0.332 | 99.0 | 101.91 (1) | <0.001 | — |
| Note: K = number of studies; Q = Cochran's Q test for heterogeneity. P for subgroup difference was derived from the Q test between subgroups. | | | | | | | |

| **Table S10. Summary of subgroup analysis for ADL at post-intervention.** | | | | | | | |
| --- | --- | --- | --- | --- | --- | --- | --- |
| **Subgroup analysis** | **K** | **SMD/MD [95% CI]** | **P** | **I² (%)** | **Q (df)** | **P (heterogeneity)** | **Subgroup difference** |
| **Overall effect** | 3 | 1.077 (-0.658, 2.813) | 0.224 | 97.5 | 79.41 (2) | <0.001 | — |
| **Duration** |  |  |  |  |  |  | — |
| short (≤4w) | 2 | 0.398 (-1.327, 2.123) | 0.651 | — | 25.32 (1) | <0.001 | — |
| **Stroke type** |  |  |  |  |  |  | Q = 4.97, df = 1, P = 0.026 |
| hemorrhagic | 1 | 2.430 (1.962, 2.898) | <0.001 | — | — | — | — |
| ischemic | 2 | 0.398 (-1.327, 2.123) | 0.651 | 96.1 | 25.32 (1) | <0.001 | — |
| **Scale** |  |  |  |  |  |  | Q = 4.97, df = 1, P = 0.026 |
| BI | 2 | 0.398 (-1.327, 2.123) | 0.651 | 96.1 | 25.32 (1) | <0.001 | — |
| others | 1 | 2.430 (1.962, 2.898) | <0.001 | — | — | — | — |
| Note: K = number of studies; Q = Cochran's Q test for heterogeneity. P for subgroup difference was derived from the Q test between subgroups. | | | | | | | |

| **Table S11. Summary of subgroup analysis for depression at follow-up.** | | | | | | | |
| --- | --- | --- | --- | --- | --- | --- | --- |
| **Subgroup analysis** | **K** | **SMD/MD [95% CI]** | **P** | **I² (%)** | **Q (df)** | **P (heterogeneity)** | **Subgroup difference** |
| **Overall effect** | 5 | -0.760 (-1.071, -0.449) | <0.001 | 53.8 | 8.66 (4) | 0.070 | — |
| **Duration** |  |  |  |  |  |  | Q = 0.34, df = 1, P = 0.559 |
| long (>4w) | 2 | -0.862 (-1.220, -0.504) | <0.001 | 0.0 | 0.06 (1) | 0.811 | — |
| short (≤4w) | 3 | -0.668 (-1.210, -0.126) | 0.016 | 76.4 | 8.46 (2) | 0.015 | — |
| **Stroke type** |  |  |  |  |  |  | Q = 1.12, df = 1, P = 0.290 |
| ischemic | 1 | -0.553 (-0.999, -0.106) | 0.015 | — | — | — | — |
| mixed | 2 | -0.862 (-1.220, -0.504) | <0.001 | 0.0 | 0.06 (1) | 0.811 | — |
| **Delivery format** |  |  |  |  |  |  | Q = 0.34, df = 1, P = 0.562 |
| group | 4 | -0.716 (-1.107, -0.325) | <0.001 | 64.6 | 8.48 (3) | 0.037 | — |
| others | 1 | -0.906 (-1.417, -0.395) | 0.001 | — | — | — | — |
| **Scale** |  |  |  |  |  |  | Q = 0.53, df = 1, P = 0.468 |
| Clinician-rated scales | 3 | -0.858 (-1.214, -0.501) | <0.001 | 51.0 | 4.08 (2) | 0.130 | — |
| Self-reported scales | 2 | -0.572 (-1.256, 0.112) | 0.101 | 67.8 | 3.11 (1) | 0.078 | — |
| Note: K = number of studies; Q = Cochran's Q test for heterogeneity. P for subgroup difference was derived from the Q test between subgroups. | | | | | | | |

| **Table S12. Summary of subgroup analysis for psychological flexibility at follow-up.** | | | | | | | |
| --- | --- | --- | --- | --- | --- | --- | --- |
| **Subgroup analysis** | **K** | **SMD/MD [95% CI]** | **P** | **I² (%)** | **Q (df)** | **P (heterogeneity)** | **Subgroup difference** |
| **Overall effect** | 3 | -4.789(-5.653,-3.926) | <0.001 | 0.0 | 1.41 (2) | 0.495 | — |
| **Duration** |  |  |  |  |  |  | Q = 1.19, df = 1, P = 0.276 |
| long (>4w) | 2 | -4.498(-5.508,-3.487) | <0.001 | 0.0 | 0.22 (1) | 0.640 | — |
| short (≤4w) | 1 | -5.580(-7.244,-3.916) | <0.001 | — | — | — | — |
| **Delivery format** |  |  |  |  |  |  | — |
| group | 3 | -4.789(-5.653,-3.926) | <0.001 | — | 1.41 (2) | 0.495 | — |
| **Scale** |  |  |  |  |  |  | — |
| AAQII | 3 | -4.789(-5.653,-3.926) | <0.001 | — | 1.41 (2) | 0.495 | — |
| Note: K = number of studies; Q = Cochran's Q test for heterogeneity. P for subgroup difference was derived from the Q test between subgroups. | | | | | | | |

| **Table S13. Meta-regression of intervention duration (random-effects, REML)** | | | | | | | | | |
| --- | --- | --- | --- | --- | --- | --- | --- | --- | --- |
| **Outcome** | k | **Covariate** | **β (95% CI)** | **SE** | **z** | **P-value** | **Residual τ²** | **Residual I² (%)** | **R² (%)** |
| Depression | 13 | Duration (weeks) | 0.081(-0.106,0.268) | 0.095 | 0.85 | 0.396 | 0.811 | 93.13 | 0.00 |
| Anxiety | 10 | Duration (weeks) | 0.106(-0.043, 0.254) | 0.076 | 1.39 | 0.164 | 0.381 | 86.46 | 11.46 |
| Psychological flexibility | 7 | Duration (weeks) | 0.368(-0.197, 0.934) | 0.288 | 1.28 | 0.202 | 8.513 | 89.56 | 11.57 |
| Note: K = number of studies; P values are two-sided Wald tests (identical to the Wald χ²(1) moderator test). Between-study variance τ² (intercept-only → with covariate): Depression 0.787 → 0.811; Anxiety 0.431 → 0.381; AAQ-II 9.627 → 8.513. Intervention duration was entered as raw weeks (not centered or standardized). | | | | | | | | | |

| **Table S14. Influence assessment: leave-one-out re-estimation of β** | | | | |
| --- | --- | --- | --- | --- |
| **Outcome** | **LOO refits** | **β range** | **P range** | **Influential study (lowest P)** |
| Depression | 13 | 0.049 to 0.138 | 0.141 to 0.618 | None (all P > 0.14) |
| Anxiety | 10 | 0.076 to 0.136 | 0.047 to 0.313 | Omitting Majumdar2019: β = 0.1299, P = 0.047 |
| Psychological flexibility | 7 | 0.079 to 0.601 | 0.073 to 0.791 | Omitting Fu2025: β = 0.6014, P = 0.073 |
| Note. LOO = leave-one-out; in each refit one study was omitted and β was re-estimated with the same REML specification. | | | | |

**Appendix 4. Forest plots for other outcomes**

**Figure S1. Forest plot of meta-analysis for the effect of ACT on psychological domain of quality of life**

**Figure S2. Forest plot of meta-analysis for the effect of ACT on hope**

**Figure S3. Forest plot of meta-analysis for the effect of ACT on resilience**

**Appendix 5. Sensitivity analysis**

**Figure S4. Sensitivity analysis on depression at post-test.**

**Figure S5. Sensitivity analysis on depression at follow-up.**

**Figure S6. Sensitivity analysis on anxiety at post-test.**

**Figure S7. Sensitivity analysis on psychological flexibility at post-test.**

**Figure S8. Sensitivity analysis on psychological flexibility at follow-up.**

**Figure S9. Sensitivity analysis on cognitive fusion at post-test.**

**Figure S10. Sensitivity analysis on quality of life (total) at post-test.**

**Figure S11. Sensitivity analysis on psychological domain of quality of life at post-test.**

**Figure S12. Sensitivity analysis on sleep at post-test.**

**Figure S13. Sensitivity analysis on activities of daily living at post-test.**

**Figure S14. Sensitivity analysis on neurological function at post-test.**

**Figure S15. Sensitivity analysis on self-efficacy at post-test.**

**Appendix 6. Publication bias test**

**Figure S16. Funnel plot of depression at post-intervention**

**Figure S17. Funnel plot of anxiety at post-intervention**

**Appendix 7. List of abbreviations**

| **List of abbreviations** | |
| --- | --- |
| ACT | Acceptance and Commitment Therapy |
| SMD | Standardised mean difference |
| MD | Mean difference |
| NR | Not Reported |
| C | Control |
| I | Intervention |
| w | Week |
| m | Month |
| BI | Barthel Index |
| CI | Confidence interval |

**Appendix 8. GRADE Assessment**

**GRADE-Evidence profile**

**Question:** ACT compared to control in patient with stroke

| **Certainty assessment** | | | | | | | **№ of patients** | | **Effect** | | **Certainty** | **Importance** |
| --- | --- | --- | --- | --- | --- | --- | --- | --- | --- | --- | --- | --- |
| **№ of studies** | **Study design** | **Risk of bias** | **Inconsistency** | **Indirectness** | **Imprecision** | **Other considerations** | **[ACT]** | **[control]** | **Relative (95% CI)** | **Absolute (95% CI)** |  |  |
| **Depression at post-intervention** | | | | | | | | | | | | |
| 16 | randomised trials | serious | serious | not serious | not serious | none | 680 | 677 | SMD -1.32  (-1.80 to -0.84) | - | ⨁⨁◯◯ Low | Critical |
| **Depression at follow-up** | | | | | | | | | | | | |
| 5 | randomised trials | serious | not serious | not serious | not serious | none | 198 | 197 | SMD -0.76  (-1.07 to -0.45) | - | ⨁⨁⨁◯ Moderate | Critical |
| **Anxiety at post-intervention** | | | | | | | | | | | | |
| 13 | randomised trials | serious | serious | not serious | not serious | none | 536 | 536 | SMD −1.36  (−1.90 to −0.82) | - | ⨁⨁◯◯  Low | Critical |
| **Anxiety at follow-up** | | | | | | | | | | | | |
| 2 | randomised trials | serious | serious | not serious | serious | none | 54 | 56 | SMD -0.51  (-1.42 to 0.40) | - | ⨁◯◯◯ Very low | Critical |
| **Psychological flexibility at post-intervention** | | | | | | | | | | | | |
| 7 | randomised trials | serious | serious | not serious | not serious | none | 334 | 333 | MD -7.23  (-9.72, -4.73) | - | ⨁⨁◯◯ Low | Critical |
| **Psychological flexibility at follow-up** | | | | | | | | | | | | |
| 3 | randomised trials | serious | serious | not serious | not serious | none | 141 | 140 | MD -4.79  (-5.65 to -3.93) | - | ⨁⨁◯◯ Low | Critical |
| **Dropout rates** | | | | | | | | | | | | |
| 16 | randomised trials | serious | not serious | not serious | not serious | not serious | 737 | 735 | RR 0.99  (0.58 to 1.69) | - | ⨁⨁◯◯ Low | Important |

**CI:** confidence interval; **SMD:** standardised mean difference; MD: mean difference

**GRADE Summary of findings**

| **Patient or population:** [patient with stroke]; **Intervention:** [ACT]; **Comparison:** [control] | | | | | | |
| --- | --- | --- | --- | --- | --- | --- |
| **Outcomes** | **Anticipated absolute effects^*^**  **(95% CI)** | | **Relative effect (95% CI)** | **№ of participants (studies)** | **Certainty of the evidence (GRADE)** | **Comments** |
|  | **Risk with [control]** | **Risk with [ACT]** |  |  |  |  |
| Depression at post-intervention | - | - | SMD -1.32  (-1.80 to -0.84) | 1,357 (16 RCTs) | ⨁⨁◯◯ Low | The evidence suggests that ACT may reduce depressive symptoms at post-intervention. |
| Depression at follow-up | - | - | SMD -0.76  (-1.07 to -0.45) | 395 (5 RCTs) | ⨁⨁⨁◯  Moderate | ACT probably reduces depression at follow-up. |
| Anxiety at post-intervention | - | - | SMD -1.36  (-1.90 to -0.82) | 1,072 (13 RCTs) | ⨁⨁◯◯ Low | The evidence suggests that ACT may reduce anxiety symptoms at post-intervention. |
| Anxiety at follow-up | - | - | SMD -0.51  (-1.42 to 0.40) | 110 (2 RCTs) | ⨁◯◯◯ Very low | The evidence is very uncertain about the effect of ACT on anxiety symptoms at follow-up. |
| Psychological flexibility at post-intervention | - | - | MD -7.23  (-9.72 to -4.73) | 667 (7 RCTs) | ⨁⨁◯◯ Low | The evidence suggests that ACT may improve psychological flexibility at post-intervention. |
| Psychological flexibility at follow-up | - | - | MD -4.79  (-5.65 to -3.93) | 281 (3 RCTs) | ⨁⨁◯◯ Low | The evidence suggests that ACT may improve psychological flexibility at follow-up. |
| Dropout rates | - | - | RR 0.99  (0.58 to 1.69) | 1,472 (16 RCTs) | ⨁⨁◯◯  Low | ACT may make little to no difference in dropout rates between ACT and control groups. |
| ***The risk in the intervention group** (and its 95% confidence interval) is based on the assumed risk in the comparison group and the **relative effect** of the intervention (and its 95% CI). **CI:** confidence interval; **SMD:** standardised mean difference; **MD**: mean difference | | | | | | |
| **GRADE Working Group grades of evidence** **High certainty:** we are very confident that the true effect lies close to that of the estimate of the effect. **Moderate certainty:** we are moderately confident in the effect estimate: the true effect is likely to be close to the estimate of the effect, but there is a possibility that it is substantially different. **Low certainty:** our confidence in the effect estimate is limited: the true effect may be substantially different from the estimate of the effect. **Very low certainty:** we have very little confidence in the effect estimate: the true effect is likely to be substantially different from the estimate of effect. | | | | | | |
